# Cross-border spread, lineage displacement and evolutionary rate estimation of rabies virus in Yunnan Province, China

**DOI:** 10.1186/s12985-017-0769-6

**Published:** 2017-06-03

**Authors:** Yuzhen Zhang, Bram Vrancken, Yun Feng, Simon Dellicour, Qiqi Yang, Weihong Yang, Yunzhi Zhang, Lu Dong, Oliver G. Pybus, Hailin Zhang, Huaiyu Tian

**Affiliations:** 1 Yunnan Institute of Endemic Diseases Control and Prevention, Yunnan Provincial Key Laboratory for Zoonosis Control and Prevention, Dali, China; 2 Department of Microbiology and Immunology, Division of Clinical and Epidemiological Virology, Rega Institute, KU Leuven - University of Leuven, Leuven, Belgium; 3 State Key Laboratory of Remote Sensing Science, College of Global Change and Earth System Science, Beijing Normal University, Beijing, China; 4 Ministry of Education Key Laboratory for Biodiversity and Ecological Engineering, College of Life Sciences, Beijing Normal University, Beijing, China; 5 Department of Zoology, University of Oxford, Oxford, UK

**Keywords:** Rabies virus, Evolutionary dynamics, Viral phylogeography, Yunnan, China, N gene

## Abstract

**Background:**

Rabies is an important but underestimated threat to public health, with most cases reported in Asia. Since 2000, a new epidemic wave of rabies has emerged in Yunnan Province, southwestern China, which borders three countries in Southeast Asia.

**Method:**

We estimated gene-specific evolutionary rates for rabies virus using available data in GenBank, then used this information to calibrate the timescale of rabies virus (RABV) spread in Asia. We used 452 publicly available geo-referenced complete nucleoprotein (N) gene sequences, including 52 RABV sequences that were recently generated from samples collected in Yunnan between 2008 and 2012.

**Results:**

The RABV N gene evolutionary rate was estimated to be 1.88 × 10^−4^ (1.37–2.41 × 10^−4^, 95% Bayesian credible interval, BCI) substitutions per site per year. Phylogenetic reconstructions show that the currently circulating RABV lineages in Yunnan result from at least seven independent introductions (95% BCI: 6–9 introductions) and represent each of the three main Asian RABV lineages, SEA-1, -2 and -3. We find that Yunnan is a sink location for the domestic spread of RABV and connects RABV epidemics in North China, South China, and Southeast Asia. Cross-border spread from southeast Asia (SEA) into South China, and intermixing of the North and South China epidemics is also well supported. The influx of RABV into Yunnan from SEA was not well-supported, likely due to the poor sampling of SEA RABV diversity. We found evidence for a lineage displacement of the Yunnan SEA-2 and -3 lineages by Yunnan SEA-1 strains, and considered whether this could be attributed to fitness differences.

**Conclusion:**

Overall, our study contributes to a better understanding of the spread of RABV that could facilitate future rabies virus control and prevention efforts.

**Electronic supplementary material:**

The online version of this article (doi:10.1186/s12985-017-0769-6) contains supplementary material, which is available to authorized users.

## Background

Rabies has one of the highest mortality rates of infectious diseases in humans, and kills almost 60,000 persons each year [1]. It is caused by the rabies virus (RABV), a virus that belongs to the genus *Lyssavirus* within the family *Rhabdoviridae*, and which is distributed almost worldwide, in particular in developing countries [2]. Asia has the highest global incidence of rabies, accounting for more than 80% of total cases [3], and China is one of the countries with the highest number of human rabies cases. Dogs are the principal vector of human rabies (98% of human cases result from dog bites) and can spread the virus across national borders and over long distances [4–6]. The RABV migration patterns can be reconstructed by analysing virus gene sequences in a phylogeographic framework, which has the potential to inform public health decisions [4].

Several major geographically-distinct clades of RABV species in nonflying mammals have been identified based on phylogenetic analyses of the RABV N gene; these are the African, cosmopolitan, Arctic-related, Asian, and Indian subcontinent clades [7, 8]. Viruses of the latter three clades circulate in Asia: (i) Indian subcontinent clade viruses are found in southern India and Sri Lanka; (ii) Arctic-related clade viruses have been reported across a large region, from Russia and central Asia to eastern Asia [9, 10]; (iii) Asian clade viruses are mainly distributed in Southeast Asia [8]. In a recent study, Guo et al. demonstrated that RABV sequences from Southeast Asia belong to three distinct clades, SEA-1 (China and Indonesia), SEA-2 (South China and the Philippines), and SEA-3 (Malaysia, Vietnam, Laos, Cambodia, and Thailand) [11]. In previous work, we sequenced 52 new RABV isolates sampled between 2008 and 2012 in Yunnan province, China. Two lineages of the clades identified in Yunnan were closely related to strains from North or South China, whilst another lineage was closely related to SEA strains [12]. Consequently, Yunnan Province was identified as a potential focal point in the region for the spread of endemic RABV across Southeast Asia. An increasing incidence of rabies cases has been reported in Yunnan, occurring in only one county in 2000 but in 77 counties in 2012 [12].

In this study, we used a Bayesian phylogenetic molecular clock approach to explore the spatio-temporal history of RABV in Yunnan, an area of China that borders three countries (Vietnam, Laos, and Myanmar) in Southeast Asia. The second aim of this study was to examine possible causes of the apparent RABV lineage displacement event that occurred in Yunnan, in which two of the three RABV lineages disappeared after 2011.

## Results

### Lineages of the three main Asian clades circulate in Yunnan

**Fig. 1:**
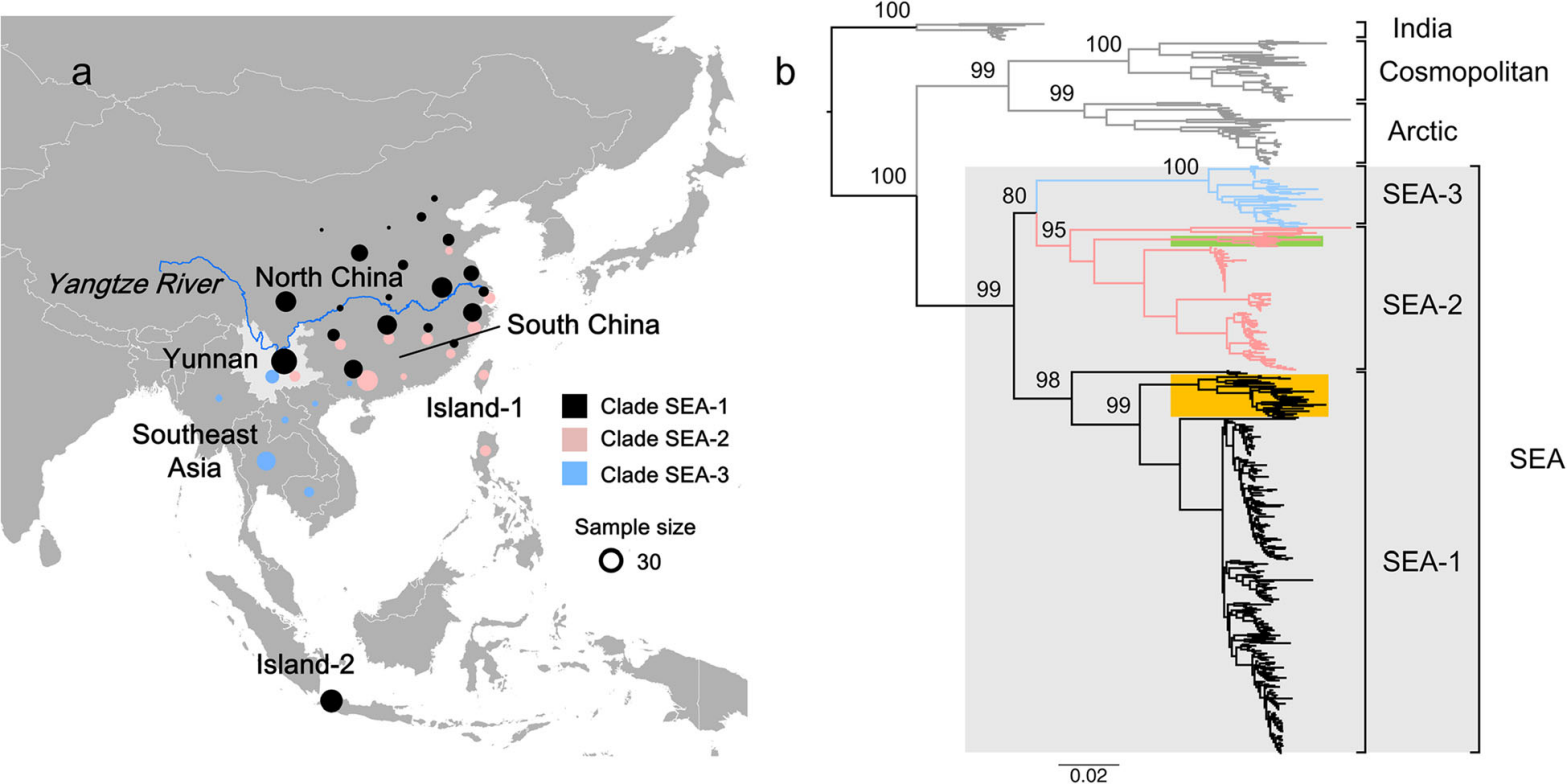
Phylogeny of Southeast Asian RABV from nonflying mammals estimated from the RABV N gene. **a** Geographic distribution of the RABV sequences used in the study. *Light gray* indicates our study area, Yunnan Province, China. North China: Hebei, Beijing, Shanxi, Shaanxi, Ningxia, Shandong, Henan, Jiangsu, Anhui, Sichuan, Hubei, and Chongqing; South China: Hunan, Jiangxi, Guizhou, Fujian, Guangxi, Shanghai, and Zhejiang; Island area-1: China Taiwan and the Philippines; Island-2: Indonesia; Southeast Asia: Myanmar, Thailand, Laos, Vietnam, and Cambodia. **b** Maximum likelihood tree of RABV estimated from N gene sequences sampled from nonflying mammals. *Numbers at each node* indicate bootstrap support values. *Scale bar* indicates nucleotide substitutions per site. Six distinct clusters are supported with strong bootstrap values: Indian subcontinent, cosmopolitan, Arctic-related, SEA-1, SEA-2, and SEA-3. Cluster in the Indonesia and Philippines is highlight in *yellow* and *green*, respectively. Full details of the dataset are given in Additional file 1: Table S1

**Fig. 2:**
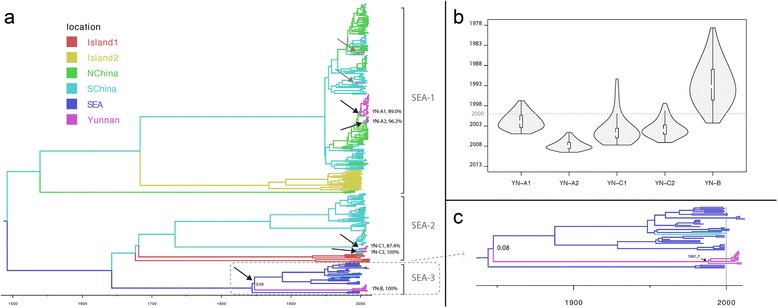
Molecular clock phylogeographic analysis of Southeast Asian RABVs using the N gene. Panel **a**: Maximum clade credibility trees of the RABV SEA clades in Asia. Branches are colored by geographic region as inferred by Bayesian phylogeographic methods and the horizontal branches are drawn on a timescale of years. The correspondance between colors and locations is as in the legend. *Black* and *grey arrows* indicate independent introduction events of RABV into Yunnan. The *grey arrows* mark single Yunnan lineages while the *black arrows* point to Yunnan clusters. Numbers next to the names of the well-supported Yunnan cluster indicate the posterior root node support for that cluster. The low posterior support for the branching structure that makes the YN-B clade an ingroup of SEA lineages in the MCC tree is given next to the corresonding node. Panel **b** Violin plot representation of the t_MRCA_’s 95%HPD estimates of the five Yunnan clusters. The *dashed line* marks the start of the most recent RABV epidemic in Yunnan. Panel **c** Enlargement of the SEA-3 clade. The *dashed grey line* marks the onset of the most recent RABV epidemic in Yunnan, which started in 2000. The median estimate of the YN-B t_MRCA_ is given next to the *black arrow* that points to the YN-B root node. The origin of >99% of all strains entering Yunnan can be traced to north and south China. Note that the rate decline in the most recent years reflects the loss of branches in the sampling time period, rather than the start of an actual tendency

Consistent with previous analyses, a phylogenetic analysis of the N gene from nonflying mammals identified six distinct clusters in Asia, which exhibited clear spatial structure: the Indian subcontinent, cosmopolitan, Arctic-related, Southeast Asia-1 (SEA-1), SEA-2, and SEA-3 lineages (Fig. 1) [7, 8, 11, 13]. The geographic locations of the different clusters of Asian RABV, SEA-1–3, are shown in Fig. 1a. Clade SEA-1, the most widespread clade across China, has been isolated in both North and South China and includes a single cluster from Indonesia (yellow highlight in Fig. 1b). Clade SEA-2 is mainly distributed in South China, South of the Yangtze River (Fig. 1a), with a single cluster in the Philippines (green highlight in Fig. 1b). Clade SEA-3 is widely distributed in Southeast Asian countries, including Myanmar, Thailand, Laos, Vietnam, Cambodia, and Yunnan Province in China. Phylogenetic analysis showed that the post-2000 Yunnan epidemic was caused by lineages from all three SEA clades (Fig. 2a).

### Temporal and spatial dynamics of RABVs in China and Southeast Asia

Our estimate of the evolutionary rate of the N gene of RABV is 1.88 × 10^−4^ (95% highest posterior density, HPD: 1.37 × 10^−4^ –2.41 × 10^−4^) substitutions per site per year. This is highly consistent with previous estimates [8, 14], and was used to calibrate the timescale of Asian RABV epidemic history.

We find strong Bayes factor support for introductions of RABV into Yunnan from North and South China (Table 1 and Additional file 1: Figure S1). Migrations from North to South China and vice versa, and from Southeast Asia to South China, are also well supported (Table 1). In contrast, the lineage migration link between Yunnan and Southeast Asian countries is less well supported but still significant (Table 1). Combined, this provides significant support for a role of Yunnan as a connector of the RABV epidemics in North and South China with those in Southeast Asia. A visualisation of the well-supported pathways of RABV migration in Asia is provided in Additional file 1: Figure S1.

**Table 1:** Overview of the significant migration links between locations

From	To	BF	PP**	BF_sub_*	PP*
North China	South China	***	39.86	***	36.25
South China	North China	***	29.31	***	30.06
**North China**	**Yunnan**	***	9.86	***	10.09
**South China**	**Yunnan**	**	9.55	**	10.46
Southeast Asia	South China	***	10.25	***	10.82
**Yunnan**	**Southeast Asia**	*	1.16	*	1.10

Migrations involving Yunnan are highlighted in boldface

*Estimated from sub-sampled data set; *MJ* Markov jumps

**PP the posterior probability for a migration event between the involved locations

The BF support for a particular type of virus movement is computed as the posterior odds over the prior odds that the rate of migration between the involved locations is non-zero. In general, 3 ≤ BF < 20 is considered as positive support, 20 ≤ BF < 150 as strong support and BF ≥ 150 as very strong support [47]. Here, the strength of support for a migration link between locations is *, ** and *** for positive, strong and very strong support respectively. Migration links involving Yunnan are highlighted in boldface

The Bayesian stochastic search variable selection (BSSVS) analysis provides information on the statistical support for each possible migration link among location pairs, but does not inform on the relative magnitude of viral movement. For this, we quantified the expected number of virus movements between locations (Markov jumps; Table 1). This shows that the bulk of virus flow is between North and South China (69.17%, Table 1). Further, almost equal amounts of virus lineage migration out of North (19.84%) or South (24.58%) China seed the RABV epidemic in Yunnan, and little more than 1% of all migration events are directed from Yunnan towards Southeast Asia. The imbalance between RABV import and export from Yunnan is reflected in the difference between the number of virus lineage movements from and to Yunnan, which is −6.1 (95%BCI: −3 to −8). The only other well-supported migration link (from southeast Asia to south China) accounts for another 10.25% of the Markov jumps. Similar patterns were obtained using a downsampled dataset with a maximum of 20 sequences per area per year (Table 1), which suggests that the results here do not appear to be driven by heterogeneous sampling.

The estimated number of independent RABV introductions into Yunnan is 7 (95% Bayesian credible interval: 6 - 9). This includes five well-supported clusters (>85% posterior support) of at least two lineages sampled in Yunnan (Fig. 2a). The SEA-3 Yunnan lineages (YN-B) are separated from the other SEA-3 strains by a long branch representing almost 140 years of evolution (Fig. 2a and c). Such a long period of undetected circulation in Yunnan is highly unlikely, and indicates that a substantial part of the SEA-3 diversity is not represented among the available data in GenBank and/or escapes detection by current surveillance programs. The t_MRCA_ estimate of the YN-B clade is 1991.7 (95%HPD: 1969.6–2003.4). Given the increased awareness after 2000 it can be assumed that RABV circulation in Yunnan was rapidly detected after that date. If RABV was detected within 1 year, the finding that 97.9% of the YN-B root node t_MRCA_ 95% HPD predates 2001 suggests that it is very likely that RABV was introduced into Yunnan from SEA on multiple occasions (at least two or three) (Fig. 2c). However, the 95%HPD interval of this clade’s t_MRCA_ extends to almost mid-2002 and the epidemic started in 2000, meaning that on the basis of these timings the possibility of one introduction event cannot be dismissed. The t_MRCA_s of the four other Yunnan clades are more recent (Fig. 2b). Given that each represents one introduction event, this shows that these lineages entered Yunnan within a rather narrow time window starting from around 2000, which is consistent with previous observations [12].

### Different sites evolved under diversifying selection in the three Asian RABV lineages

The overall d_N_/d_S_ ratio for the N gene of RABV in Yunnan was low (0.047), suggesting that strong purifying selection has been the main evolutionary pressure acting on RABV [15]. Interestingly, the branch-specific d_N_/d_S_ values for RABV clades SEA-2 and SEA-3 in Yunnan were higher (0.056) than that for branches in clade SEA-1 (0.009). We applied two likelihood ratio tests (LRTs) that compare codon substitution model M7 (no positive selection) against model M8 (positive selection allowed) in order to detect positive selection at the 5% level. For the SEA-1 clade, the LRT statistic comparing M7 and M8 was 2 In*L* = 2 × (In*L*_M8_ - In*L*_M7_) = 11.28; thus the null hypothesis of no positive selection could be rejected. For clades SEA-2 and SEA-3, they were 2 In*L* = 2 × (In*L*_M8_ - In*L*_M7_) = 185.50. Some sites in the N gene (sites 90, 128, 135, 379, 397, and 426) were identified with both the M8 model in CODEML and the FEL method in HYPHY as being positively selected (Table 2). Overall, these results are indicative of positive selection acting at these sites. We then compared the amino acid sites that vary between the SEA-1, SEA-2, and SEA-3 clades (Table 3). The SEA-2 lineages carry six different N-gene AAs and the SEA-3 strains seven AAs, when compared to SEA-1 lineages. Two of these differences with respect to SEA-1 strains are shared between the SEA-2 and SEA-3 lineages: S42T and S397G (Table 3). It is of note that the SEA-3 AA residues at positions within linear epitopes (sites 375 and 379) are different from those in SEA-1 strains, in particular because both sites were subject to diversifying selection according to at least one method (Tables 2 and 3). The SEA-2 lineages, on the other hand, share their AA residues at these epitope positions with SEA-1 strains.

**Table 2:** Positively selected sites in the N gene in Yunnan

	M7	M8			
Clade	InL	InL	*χ* ^2^	*P*	Branchd_N_/d_S_
SEA-1	−2551.03	−2545.39	5.63	0.06	0.009
SEA-2 & SEA-3	−4395.09	−4302.34	92.75	<0.01	0.056

**Table 3:** Key variable sites between the clade SEA-1, clade SEA-2, and clade SEA-3 viruses, Yunnan Province

	Amino acid position in the N protein										
Clade	42	90^b,c^	110	128^b,c^	135^b,c^	157	254	375^b,a^	379^b,c,a^	397	426^b,c^
SEA-1	S	T	E	L	S	N	R	T	L	S	S
SEA-2	T	N	D	L	S	S	K	T	L	G	S
SEA-3	T	T	E	V	A	N	R	M	V	G	A

^a^Linear epitope (antigenic site IV)

^b^Positively selected sites were identified with Bayes empirical Bayes (BEB) analyses in CODEML

^c^Positively selected sites that were identified with fixed-effect likelihood (FEL) method implemented in HYPHY

## Discussion

Rabies remains one of the most dangerous but neglected diseases in developing countries, especially in Asia and Africa, where >95% of human cases occur [16]. In this study, we used Bayesian phylodynamic methods to investigate the spatiotemporal aspects of RABV evolution in Asia with a focus on the role of the Yunnan province of China in the regional epidemic history of the virus. For this we used publicly available time-annotated near complete viral genomes, as well as N gene sequences whose locations and dates of sampling were known. The former were used to estimate gene-specific evolutionary rates, which allowed us to put a timescale on virus migration history.

Results from the evolutionary reconstructions show that the reemergent RABV lineages in Yunnan since 2000 contain representatives of the three main Southeast Asian clades (Fig. 2a). Interestingly, no SEA-3 strains were detected after 2010 and only one SEA-2 lineage was found in 2011 in Yunnan, even though the surveillance efforts covered the same geographical area throughout the entire sampling period (2008–2012) [12]. Conditioning on the representativeness of our sampling for the Yunnan RABV epidemic, this indicates that the YN-B and YN-C strains were unable to maintain sustained transmission chains. This can either be a stochastic variation or represent differences in adaptation to the epidemiological environment in Yunnan, which might enable SEA-1 to outcompete the SEA-2 and SEA-3 strains. The consensus amino acids of two antibody-binding sites that have been positively selected in the SEA-3 clade differ from those in SEA-1 lineages, which is consistent with the hypothesis that the apparent lineage displacement of SEA-3 by SEA-1 lineages is linked to differences in “pre-adaptation” [17] to the Yunnan epidemiological environment. The AA consensus residues at the antibody-binding sites are shared between SEA-1 and SEA-2 strains, which means that fitness differences linked to immunogenic properties cannot explain the apparent SEA-2 lineage displacement. Thus, a mixture of neutral lineage dynamics (the disappearance of SEA-2) and selection (the disappearance of SEA-3) may have been at play. More research is, however, needed to test and validate the hypothesis of a relationship between lineage displacement and fitness differences. For example, the SEA-2 and SEA-3 clades harbor the same consensus residue difference with respect to the SEA-1 strains at positions 42 and 397, but there is no information about the potential impact of this on the virus’s replicative fitness. Finally, this evaluation is limited to the N gene, and cannot detect an impact of AA substitutions in other genes.

The lineage migration analyses revealed that there is no well-supported RABV migration from China to other countries other than migration via Yunnan. Complementary to its role as a funnel for RABV migration from north and south China to southeast Asia, Yunnan also acts as a sink region for domestic RABVs. Previous work has associated RABV dissemination with patterns of animal trade and dog relocation [18–21]. However, our results also show that a completely effective screening at best would have prevented only 1 strain from starting a chain of infections every two years (Additional file 1: Figure S3). Even though these estimates represent a lower boundary, the large number of screenings required to detect a positive case indicates that alternative intervention efforts based on early detection and large-scale preventive vaccination campaigns likely are more effective in containing RABV spread.

Import of RABV into south China from southeast Asia was the only well-supported immigration link into China. The temporal dynamics of the YN-B lineage however, indicates that the sample of the SEA-3 genetic diversity in GenBank likely is incomplete. This, together with the frequent clustering of the YN-B clade as an outgroup to the other SEA-3 strains (Figure 2a and c), likely accounts for the failure of the phylogeographic analysis to detect a well-supported link for RABV import into China from SEA via Yunnan. It also follows that the high ratio of expected within-country movements versus international movements (Table 1), represents an upper limit. Nevertheless, it is clear that in China within-country circulation accounts for more infections than virus importation.

## Conclusion

This work provides new insights into the spatial diffusion patterns of RABV across large-scale regions in Asia. The main limitation of this work is the lack of a genome-wide perspective on RABV evolution, which is a consequence of the lack of large numbers of publicly available RABV sequence information for genes other than the N gene. However, as recombination in RABV is rare, our N-gene based results may be representative of the whole genome, similar to that seen for hepatitis C viruses [22].

## Methods

### Sample collection

Between 2008 and 2012, brain tissue was collected from dogs and deceased patients, and saliva and cerebrospinal fluid samples from surviving patients in Yunnan Province, China, as described previously [12]. All samples were tested for RABV antigen, and the viral genes of the rabies-positive samples were sequenced. Viral RNA was extracted by using TRIzol Reagent (Invitrogen, Carlsbad, CA, USA) and used as template for cDNA with Ready-To-Go You-Prime First-Strand Beads (GE Healthcare, Piscataway, NJ, USA). Complete N gene sequences were obtained by using primers specific for this gene as described [19, 23]. PCR products were purified by using the QIAquick PCR Purification Kit (QIAGEN, Hilden, Germany) and sequenced. A total of 52 complete RABV nucleoprotein (N) gene sequences were obtained using primers specific for this gene, as described elsewhere [12, 19, 23].

The nucleotide sequences of the N genes of all available RABVs sampled from nonflying mammals in Asia were extracted from GenBank. The sequences were aligned using the MAFFT program [24]. A total of 452 N sequences (the entire N-coding sequence, 1350 nt long), including our newly acquired sequences, were analyzed in this study (Additional file 1: Table S1). Preliminary maximum likelihood trees for data verification (GTR substitution model with 10,000 bootstrap replicates) were estimated using FASTTREE v2.1.4 [25]. Additional file 1: Table S1 shows the accession numbers, dates of isolation, and origins of the included isolates. Potential recombinant sequences identified by the Recombination Detection Program [26] were excluded from further analyses (GenBank accession numbers FJ712195, JN974825, FJ712196, and EU159363).

### Evolutionary rate estimation for N gene sequences

Because preliminary analysis indicated that the N-gene dataset lacked a clear temporal signal we first estimated gene-specific evolutionary rates using the available data in GenBank to specify a suitable prior distribution for the evolutionary rate parameter in subsequent time-scaled phylogenetic analyses. The (near) full genomes (sequence lengths >9550 nt, Additional file 1: Table S2) for which the collection date is specified in GenBank almost all represent the completely N, G, M1, M2 and L genes (see Additional file 1: Figure S4) and were aligned with MAFFT [27]. After removing two 1931 and three 1956 sequences from cell culture propagated lineages, we followed Vrancken et al. [28] to obtain a downsampled dataset with clear temporal signal. Plotting the root-to-tip genetic divergence against sampling time [29] revealed one outlying sequence that was consequently removed. The resulting 93 taxon dataset with samples collected between 1950 and 2015 showed no signal for recombination with the Phi-test [30] nor for confounding between temporal and genetic structure [31]. Reassuringly, the correlation between accrued genetic divergence and sampling time (rho = 0.27) as measured with linear regression was significant (p < 0.01 against a null distribution created from 1000 randomisations) [31]. The regression was performed on a maximum likelihood tree estimated using the default PhyML settings in Seaview [32, 33]. The sampling time distribution of the final dataset is given in Additional file 1: Table S3.

To obtain gene-specific substitution rate estimates we follow the strategy of Al-Qahtani et al. [34]. Rates were estimated with an uncorrelated clock model with the rates drawn from a lognormal distribution [35] and a relative rate approach was used to allow for among gene rate variation [13]. The SkyGrid model was specified as a flexible prior on the tree [36]. A HKY + Gamma substitution model was fitted to each partition and information on the substitution processes was shared across the gene partitions through hierarchical prior distribution specification [37]. As a final check we also conducted a date-randomisation test as implemented in BEAST [38] to compare the rate estimates obtained under the tip-date calibrated clock model with a corresponding null distribution. Following Firth et al [39] we accepted the veracity of the temporal signal because the mean of the substitution rate estimate obtained from the empirical sequence sampling dates falls outside the 95% highest posterior density interval (95% HPD) of the null distribution of rates from the randomised data. The gene-specific evolutionary rate estimate for the N-gene was used to specify a normal prior distribution on the mean clock rate for our RABV dataset. Specifically, the mean of the latter was set to the mean of the N-gene rate estimate, and the standard deviation was set so that the 2.5 and 97.5 percentiles of the normal distribution correspond to the lower and upper boundaries of the N-gene rate estimate’s 95% HPD.

### Phylogeography of the RABV N gene

To mitigate possible biases arising from the uneven sample size at each location (Additional file 1: Figure S1) the sequences of the N gene were grouped into six geographic regions: North China (including Hebei, Beijing, Shanxi, Shaanxi, Ningxia, Shandong, Henan, Jiangsu, Anhui, Sichuan, Hubei, and Chongqing, *n* = 128), South China (including Hunan, Jiangxi, Guizhou, Fujian, Guangxi, Shanghai, and Zhejiang, *n* = 180), Yunnan Province (*n* = 63), Island area-1 (China Taiwan and the Philippines, *n* = 13), Island area-2 (Indonesia, *n* = 33), and Southeast Asia (Myanmar, Thailand, Laos, Vietnam, and Cambodia, *n* = 35) (Additional file 1: Figure S2). In order to create a more even spatio-temporal sampling distribution, we also randomly subsampled the large sequence datasets by area and sampling time. At most 20 sequences were sampled per area and per year. After sub-sampling the total number of sequences analyzed here was 401 over a total of 51 years (Additional file 1: Table S4).

Discrete phylogenetic analyses of RABV lineage movement among these six geographic regions were performed under an asymmetric model of among-location transition [40], estimated using the MCMC approach implemented in BEAST v. 1.8.2 (http://beast.bio.ed.ac.uk/). A Bayesian skygrid coalescent model [36], the SRD06 model of nucleotide substitution [41], and a relaxed (uncorrelated log-normal) molecular clock model were used in this analysis. A Bayesian stochastic search variable selection (BSSVS) approach was used to identify the best-supported among-location movement rates and Spread3 [42] was used to calculated Bayes factor (BF) support for the transition rates. We also estimated the number of transitions among locations (Markov jumps) [43]. Three chains of 2.5 × 10^8^ steps, subsampled every 50,000th generation, were combined after the removal of the 10% burn-in. The convergence of MCMC output of all parameters was inspected with Tracer v1.5.

### Analysis of selection pressures

We used a phylogenetic codon substitution model approach, implemented in the CODEML program of the PAML package [44], to detect positive selection in RABV in Yunnan Province. The numbers of nonsynonymous (d_N_) and synonymous substitutions (d_S_) per site and their ratio (d_N_/d_S_) were estimated for each codon of RABV from nonflying mammals in Yunnan, using the M7 and M8 models [45]. Putative positively selected sites were also identified using the fixed-effect likelihood (FEL) method implemented in HYPHY [46]. An amino acid position was considered to be under positive selection if its d_N_/d_S_ ratio was > 1 and the likelihood ratio test (LRT) had a significance level of *P* < 0.05. An overall dN/dS ratio was also calculated using all branches (one-ratio model) for clades SEA-1–3. To examine selection pressures in more detail, separate dN/dS values were estimated for branches of the same phylogeny (two-ratio model).

## Additional file

Additional file 1:
**Figure S1.** Raw data of RABV N gene sequences in each location in each year in Southeast Asia. **Figure S2.** RABV N gene sequences in each area in each year. The color spectrum shows the number of sequences from each year and area, from green (low numbers of sequences) to dark red (high numbers of sequences). The data set contained 452 RABV sequences. **Figure S3.** Histogram showing the temporal trend of the expected number of RABV introductions from North and South China into Yunnan. **Figure S4.** Plot showing the number of sequences that cover each position in the RABV genome. The coverage low around position 4945 corresponds to a string of six guanines that is only 5 guanines long in many strains. **Table S1.** Sequences used in this study. **Table S2.** Time-annotated (near) full genome sequences used for estimating evolutionary rates. **Table S3.** The sampling time distribution of the final dataset. **Table S4.** Subsampled sequences used in phylogeographical analysis. (DOCX 332 kb)

## Abbreviations

BSSVS: Bayesian stochastic search variable selection
FEL: Fixed-effect likelihood
HPD: Highest posterior density
LRT: Likelihood ratio test
RABV: Rabies viruses
