# Supplementary material for: Cross-border spread, lineage displacement and evolutionary rate estimation of rabies virus in Yunnan Province, China
Source: Virol J. 2017 Jun 3;14:102. doi: 10.1186/s12985-017-0769-6 (PMC5457581; doi:10.1186/s12985-017-0769-6)
Supplement: Additional file 1: — Figure S1. Raw data of RABV N gene sequences in each location in each year in Southeast Asia. Figure S2. RABV N gene sequences in each area in each year. The color spectrum shows the number of sequences from each year and area, from green (low numbers of sequences) to dark red (high numbers of sequences). The data set contained 452 RABV sequences. Figure S3. Histogram showing the temporal trend of the expected number of RABV introductions from North and South China into Yunnan. Figure S4. Plot showing the number of sequences that cover each position in the RABV genome. The coverage low around position 4945 corresponds to a string of six guanines that is only 5 guanines long in many strains. Table S1. Sequences used in this study. Table S2. Time-annotated (near) full genome sequences used for estimating evolutionary rates. Table S3. The sampling time distribution of the final dataset. Table S4. Subsampled sequences used in phylogeographical analysis. (DOCX 332 kb) [file 12985_2017_769_MOESM1_ESM.docx]

**Additional file 1**


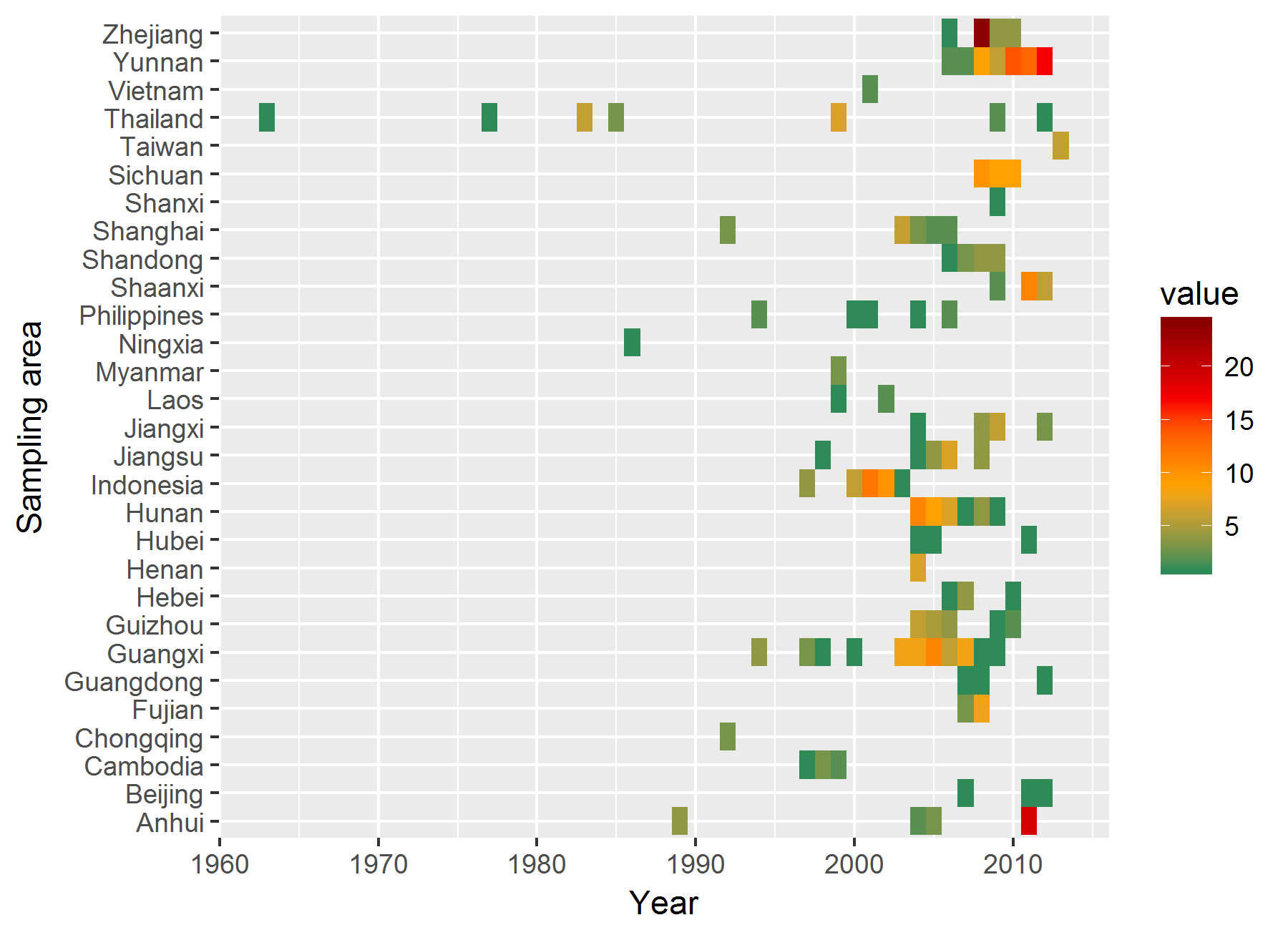


**Figure S1.** Raw data of RABV N gene sequences in each location in each year in Southeast Asia.


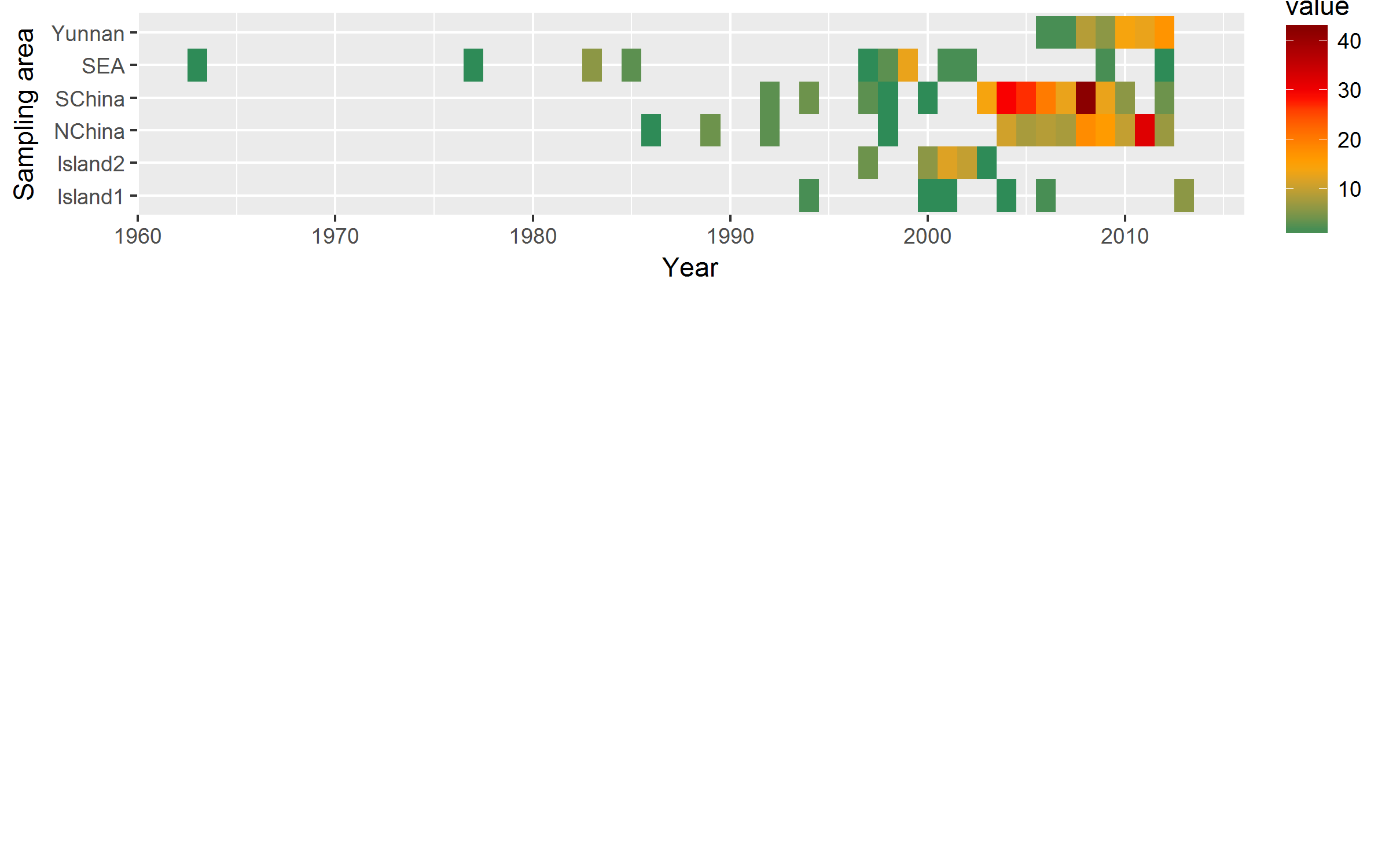


**Figure S2.** RABV N gene sequences in each area in each year. The color spectrum shows the number of sequences from each year and area, from green (low numbers of sequences) to dark red (high numbers of sequences). The data set contained 452 RABV sequences.


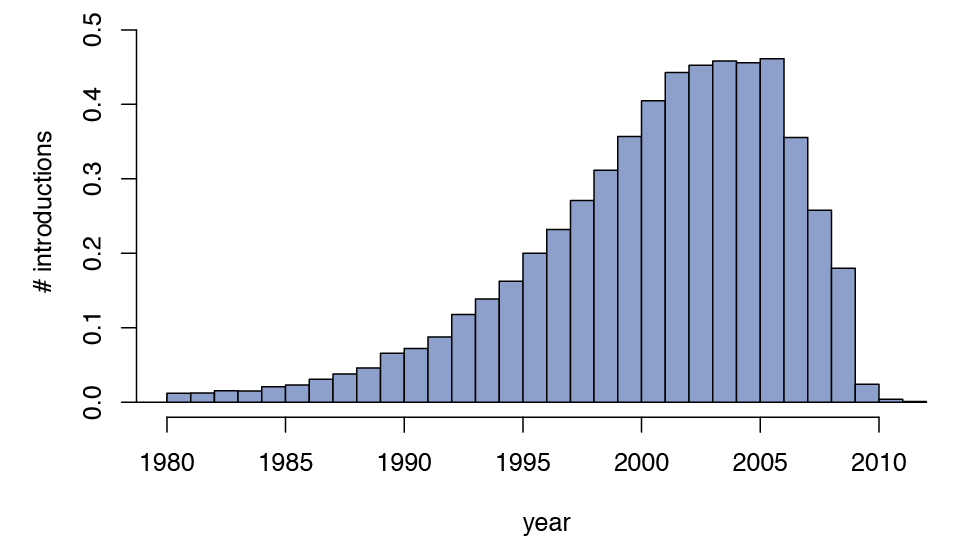


Figure S3. Histogram showing the temporal trend of the expected number of RABV introductions from North and South China into Yunnan.


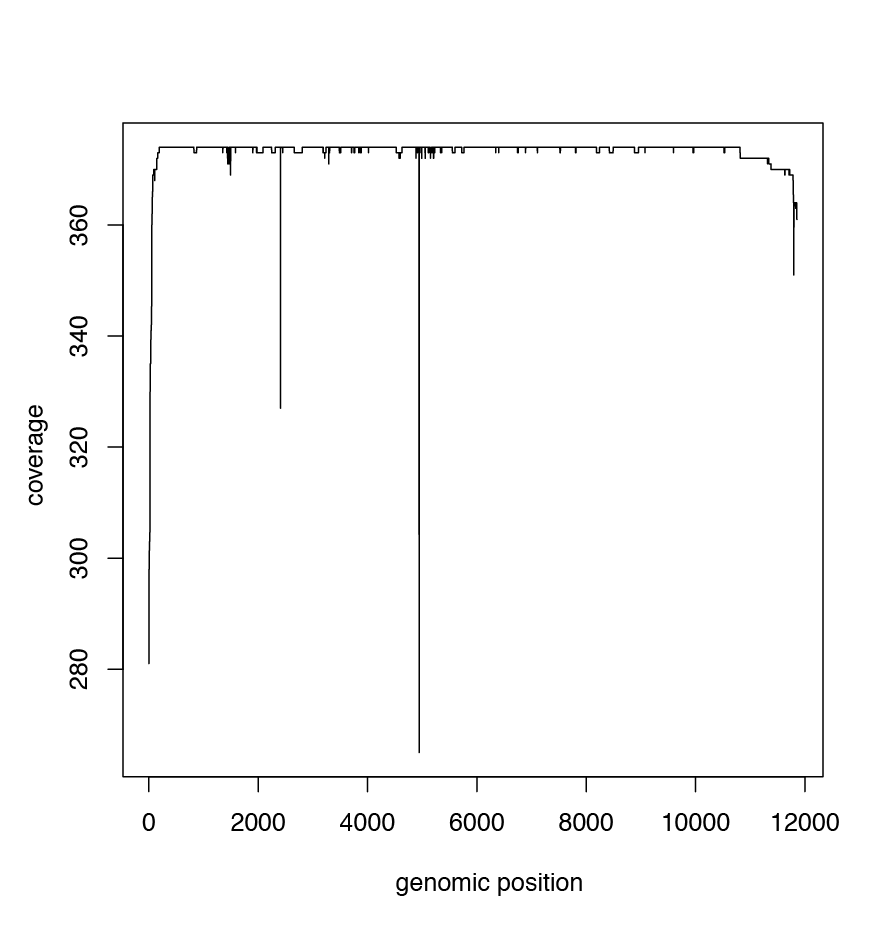


Figure S4. Plot showing the number of sequences that cover each position in the RABV genome. The coverage low around position 4945 corresponds to a string of six guanines that is only 5 guanines long in many strains.

**Table S1** Sequences used in this study

| Accession number | Year | Location | Area | Host |
| --- | --- | --- | --- | --- |
| AY218996 | 1999 | Thailand | SEA | Canine |
| AY218997 | 1999 | Thailand | SEA | Canine |
| AY218998 | 1999 | Thailand | SEA | Canine |
| AY218999 | 1999 | Thailand | SEA | Human |
| AY219000 | 1999 | Thailand | SEA | Human |
| AY219001 | 1999 | Thailand | SEA | Human |
| AY219002 | 1999 | Thailand | SEA | Human |
| AB154208 | 2001 | Indonesia | Island2 | Cat |
| AB154209 | 2002 | Indonesia | Island2 | Cat |
| AB154210 | 2001 | Indonesia | Island2 | Cat |
| AB154211 | 2002 | Indonesia | Island2 | Cat |
| AB154212 | 2001 | Indonesia | Island2 | Cattle |
| AB154213 | 2001 | Indonesia | Island2 | Civet cat |
| AB154214 | 2001 | Indonesia | Island2 | Deer |
| AB154215 | 2001 | Indonesia | Island2 | Canine |
| AB154216 | 2002 | Indonesia | Island2 | Canine |
| AB154220 | 1997 | Indonesia | Island2 | Canine |
| AB154221 | 2000 | Indonesia | Island2 | Canine |
| AB154222 | 2001 | Indonesia | Island2 | Canine |
| AB154223 | 1997 | Indonesia | Island2 | Canine |
| AB154224 | 2002 | Indonesia | Island2 | Monkey |
| AB154225 | 2000 | Indonesia | Island2 | Canine |
| AB154226 | 2000 | Indonesia | Island2 | Canine |
| AB154227 | 2000 | Indonesia | Island2 | Canine |
| AB154228 | 2001 | Indonesia | Island2 | Canine |
| AB154229 | 2002 | Indonesia | Island2 | Canine |
| AB154230 | 2002 | Indonesia | Island2 | Canine |
| AB154231 | 2002 | Indonesia | Island2 | Canine |
| AB154232 | 2002 | Indonesia | Island2 | Canine |
| AB154233 | 1997 | Indonesia | Island2 | Canine |
| AB154234 | 2000 | Indonesia | Island2 | Canine |
| AB154235 | 2000 | Indonesia | Island2 | Canine |
| AB154237 | 2001 | Indonesia | Island2 | Canine |
| AB154238 | 2001 | Indonesia | Island2 | Canine |
| AB154239 | 2001 | Indonesia | Island2 | Canine |
| AB154240 | 2002 | Indonesia | Island2 | Canine |
| AB154241 | 1997 | Indonesia | Island2 | Canine |
| AB154242 | 2001 | Indonesia | Island2 | Tiger |
| AB154243 | 2002 | Indonesia | Island2 | Canine |
| AB178892 | 1985 | Thailand | SEA | Canine |
| AB178893 | 1985 | Thailand | SEA | Canine |
| AB178894 | 1985 | Thailand | SEA | Canine |
| AB178895 | 1977 | Thailand | SEA | Canine |
| AB178896 | 1963 | Thailand | SEA | Canine |
| DQ496219 | 2006 | Hunan | SChina | Pig |
| DQ515993 | 2006 | Hunan | SChina | Canine |
| DQ666287 | 2004 | Guangxi | SChina | Canine |
| DQ666289 | 2004 | Guizhou | SChina | Canine |
| DQ666290 | 2004 | Guizhou | SChina | Canine |
| DQ666291 | 2004 | Guizhou | SChina | Canine |
| DQ666294 | 2004 | Guizhou | SChina | Canine |
| DQ666295 | 2004 | Guizhou | SChina | Canine |
| DQ666296 | 2004 | Guizhou | SChina | Canine |
| DQ666297 | 2004 | Henan | NChina | Canine |
| DQ666298 | 2004 | Henan | NChina | Canine |
| DQ666299 | 2004 | Henan | NChina | Canine |
| DQ666300 | 2004 | Henan | NChina | Canine |
| DQ666302 | 2004 | Henan | NChina | Canine |
| DQ666304 | 2004 | Henan | NChina | Canine |
| DQ666306 | 2004 | Henan | NChina | Canine |
| DQ666307 | 2004 | Hunan | SChina | Canine |
| DQ666308 | 2004 | Hunan | SChina | Canine |
| DQ666309 | 2004 | Hunan | SChina | Canine |
| DQ666311 | 2004 | Hunan | SChina | Canine |
| DQ666312 | 2004 | Hunan | SChina | Canine |
| DQ666314 | 2004 | Hunan | SChina | Canine |
| DQ666315 | 2004 | Hunan | SChina | Canine |
| DQ666316 | 2004 | Hunan | SChina | Canine |
| DQ666317 | 2004 | Hunan | SChina | Canine |
| DQ666318 | 2004 | Hunan | SChina | Canine |
| DQ666319 | 2004 | Hunan | SChina | Canine |
| DQ666321 | 2004 | Jiangsu | NChina | Canine |
| DQ866105 | 2004 | Guangxi | SChina | Canine |
| DQ866106 | 2003 | Guangxi | SChina | Canine |
| DQ866107 | 2003 | Guangxi | SChina | Canine |
| DQ866108 | 2003 | Guangxi | SChina | Canine |
| DQ866109 | 2003 | Guangxi | SChina | Canine |
| DQ866110 | 2004 | Guangxi | SChina | Canine |
| DQ866111 | 2000 | Guangxi | SChina | Canine |
| DQ866112 | 2004 | Guangxi | SChina | Canine |
| DQ866113 | 2003 | Guangxi | SChina | Canine |
| DQ866114 | 2004 | Guangxi | SChina | Canine |
| DQ866115 | 2003 | Guangxi | SChina | Canine |
| DQ866116 | 2003 | Guangxi | SChina | Canine |
| DQ866117 | 2004 | Guangxi | SChina | Canine |
| DQ866118 | 2005 | Guangxi | SChina | Canine |
| DQ866119 | 2005 | Guangxi | SChina | Canine |
| DQ866120 | 2005 | Guangxi | SChina | Canine |
| DQ866121 | 2005 | Guangxi | SChina | Canine |
| AB294348 | 2005 | Guangxi | SChina | Canine |
| AB294349 | 2005 | Guangxi | SChina | Wild pig |
| AB294350 | 2004 | Guangxi | SChina | Canine |
| AB294351 | 2004 | Guangxi | SChina | Canine |
| AB294352 | 2003 | Guangxi | SChina | Canine |
| AB294355 | 2005 | Guangxi | SChina | Bovine |
| EF611081 | 2004 | Hubei | NChina | Buffalo |
| EU008919 | 2005 | Hunan | SChina | Canine |
| EU008920 | 2005 | Hunan | SChina | Canine |
| EU008921 | 2005 | Hunan | SChina | Canine |
| EU008922 | 2005 | Hunan | SChina | Canine |
| EU008923 | 2005 | Hunan | SChina | Canine |
| EU095330 | 2006 | Yunnan | Yunnan | Canine |
| EU086164 | 1999 | Myanmar | SEA | Canine |
| EU086165 | 1999 | Myanmar | SEA | Canine |
| EU086166 | 1999 | Myanmar | SEA | Canine |
| EU086167 | 1999 | Cambodia | SEA | Canine |
| EU086168 | 1998 | Cambodia | SEA | Canine |
| EU086169 | 1998 | Cambodia | SEA | Canine |
| EU086170 | 1997 | Cambodia | SEA | Canine |
| EU086171 | 1999 | Cambodia | SEA | Canine |
| EU086172 | 1998 | Cambodia | SEA | Canine |
| EU086173 | 1998 | Jiangsu | NChina | Canine |
| EU086175 | 1997 | Guangxi | SChina | Canine |
| EU086176 | 1992 | Shanghai | SChina | Human |
| EU086182 | 1994 | Guangxi | SChina | Canine |
| EU086183 | 1994 | Guangxi | SChina | Canine |
| EU086185 | 1992 | Chongqing | NChina | Human |
| EU086188 | 2004 | Jiangxi | SChina | Canine |
| EU086192 | 2003 | Indonesia | Island2 | Canine |
| EU086193 | 1999 | Laos | SEA | Canine |
| EU086194 | 2002 | Laos | SEA | Canine |
| EU086195 | 2002 | Laos | SEA | Canine |
| EU086200 | 1994 | Philippines | Island1 | Canine |
| EU086202 | 1994 | Philippines | Island1 | Canine |
| EU086203 | 2000 | Philippines | Island1 | Human |
| EU086204 | 2001 | Philippines | Island1 | Homo sapiens |
| EU086205 | 2004 | Philippines | Island1 | Homo sapiens |
| EU086206 | 1983 | Thailand | SEA | Human |
| EU086207 | 1983 | Thailand | SEA | Human |
| EU086208 | 1983 | Thailand | SEA | Human |
| EU086209 | 2001 | Vietnam | SEA | Canine |
| EU086210 | 2001 | Vietnam | SEA | Canine |
| EU267777 | 2007 | Hebei | NChina | Human |
| EU159368 | 2004 | Anhui | NChina | Canine |
| EU159380 | 2005 | Hubei | NChina | Canine |
| EU159382 | 2005 | Jiangsu | NChina | Canine |
| EU159384 | 2005 | Jiangsu | NChina | Canine |
| EU159385 | 1997 | Guangxi | SChina | Canine |
| EU159388 | 1992 | Chongqing | NChina | Canine |
| EU159390 | 1998 | Guangxi | SChina | Canine |
| EU159392 | 1992 | Shanghai | SChina | Human |
| EU159393 | 1992 | Shanghai | SChina | Canine |
| EU159394 | 2006 | Shanghai | SChina | Canine |
| EU159395 | 2006 | Guizhou | SChina | Canine |
| EU159397 | 2006 | Zhejiang | SChina | Canine |
| EU159399 | 1994 | Guangxi | SChina | Canine |
| EU159400 | 1989 | Anhui | NChina | Canine |
| EU159401 | 1989 | Anhui | NChina | Canine |
| EU275243 | 2006 | Yunnan | Yunnan | Canine |
| EU275244 | 2007 | Yunnan | Yunnan | Canine |
| EU275245 | 2007 | Yunnan | Yunnan | Canine |
| EU293111 | 1983 | Thailand | SEA | Human |
| EU293121 | 1983 | Thailand | SEA | Human |
| EU549783 | 2006 | Hebei | NChina | Canine |
| EU700031 | 2007 | Beijing | NChina | Homo sapiens |
| EU700032 | 2008 | Zhejiang | SChina | Human |
| EU828651 | 2007 | Hebei | NChina | Canine |
| EU828653 | 2007 | Guangdong | SChina | Canine |
| EU828655 | 2007 | Hebei | NChina | Canine |
| EU828657 | 2007 | Hebei | NChina | Canine |
| EU643590 | 2006 | Hunan | SChina | Canine |
| FJ561726 | 2008 | Fujian | SChina | Canine |
| FJ561727 | 2008 | Fujian | SChina | Canine |
| FJ561728 | 2008 | Fujian | SChina | Canine |
| FJ598135 | 2008 | Zhejiang | SChina | Ferret badger |
| FJ712193 | 2008 | Zhejiang | SChina | Canine |
| FJ712194 | 2008 | Zhejiang | SChina | Canine |
| FJ719751 | 2008 | Jiangxi | SChina | Ferret badger |
| FJ719753 | 2008 | Jiangxi | SChina | Ferret badger |
| FJ719755 | 2008 | Jiangxi | SChina | Ferret badger |
| FJ719760 | 2008 | Zhejiang | SChina | Canine |
| FJ866827 | 2008 | Fujian | SChina | Canine |
| FJ866828 | 2008 | Fujian | SChina | Canine |
| FJ866829 | 2007 | Fujian | SChina | Canine |
| FJ866830 | 2007 | Fujian | SChina | Canine |
| FJ866831 | 2007 | Fujian | SChina | Canine |
| FJ866835 | 2008 | Fujian | SChina | Canine |
| FJ866836 | 2008 | Fujian | SChina | Canine |
| FJ594278 | 1997 | Guangxi | SChina | Canine |
| GQ303555 | 2009 | Thailand | SEA | Homo sapiens |
| GQ303556 | 2009 | Thailand | SEA | Canine |
| GU233765 | 2009 | Jiangxi | SChina | Ferret badger |
| GU345746 | 1992 | Chongqing | NChina | Canine |
| GU345747 | 1986 | Ningxia | NChina | Homo sapiens |
| GU345748 | 2006 | Shanghai | SChina | Canine |
| GU358653 | 1994 | Guangxi | SChina | Canine |
| GU647092 | 2008 | Jiangxi | SChina | Ferret badger |
| GU591790 | 2009 | Shaanxi | NChina | Canine |
| GU591792 | 2009 | Sichuan | NChina | Canine |
| GU992307 | 1983 | Thailand | SEA | Canine |
| HM756692 | 2008 | Hunan | SChina | Canine |
| HM486348 | 2005 | Jiangsu | NChina | Canine |
| HM486349 | 2006 | Jiangsu | NChina | Canine |
| HM486350 | 2006 | Jiangsu | NChina | Canine |
| HM486351 | 2006 | Jiangsu | NChina | Canine |
| HM486352 | 2006 | Jiangsu | NChina | Canine |
| HM486353 | 2005 | Jiangsu | NChina | Canine |
| HM486354 | 2006 | Jiangsu | NChina | Canine |
| HM486355 | 2004 | Shanghai | SChina | Canine |
| HM486356 | 2004 | Shanghai | SChina | Canine |
| HM486357 | 2003 | Shanghai | SChina | Canine |
| HM486358 | 2003 | Shanghai | SChina | Canine |
| HM486359 | 2003 | Shanghai | SChina | Canine |
| HM486360 | 2005 | Anhui | NChina | Canine |
| HM486361 | 2005 | Anhui | NChina | Canine |
| HM486362 | 2005 | Anhui | NChina | Canine |
| HM486363 | 2006 | Jiangsu | NChina | Canine |
| HM486364 | 2006 | Jiangsu | NChina | Canine |
| HM486365 | 2006 | Guangxi | SChina | Canine |
| HM486366 | 2005 | Guangxi | SChina | Canine |
| HM486367 | 2005 | Guizhou | SChina | Canine |
| HM486368 | 2005 | Guizhou | SChina | Canine |
| HM486369 | 2006 | Guizhou | SChina | Canine |
| HM486370 | 2005 | Guangxi | SChina | Canine |
| HM486371 | 2005 | Guizhou | SChina | Canine |
| HM486372 | 2006 | Guizhou | SChina | Canine |
| HM486373 | 2008 | Zhejiang | SChina | Canine |
| HM486374 | 2008 | Zhejiang | SChina | Canine |
| HM486375 | 2008 | Zhejiang | SChina | Canine |
| HM486376 | 2008 | Shandong | NChina | Canine |
| HM486377 | 2008 | Shandong | NChina | Canine |
| HM486378 | 2007 | Shandong | NChina | Canine |
| HM486379 | 2007 | Shandong | NChina | Canine |
| HM486380 | 2006 | Shandong | NChina | Canine |
| HM486381 | 2007 | Shandong | NChina | Canine |
| GQ472468 | 2007 | Guangxi | SChina | Canine |
| GQ472469 | 2007 | Guangxi | SChina | Canine |
| GQ472470 | 2007 | Guangxi | SChina | Canine |
| GQ472471 | 2007 | Guangxi | SChina | Canine |
| GQ472472 | 2007 | Guangxi | SChina | Canine |
| GQ472473 | 2007 | Guangxi | SChina | Canine |
| GQ472474 | 2007 | Guangxi | SChina | Canine |
| GQ472475 | 2005 | Guangxi | SChina | Canine |
| GQ472476 | 2006 | Guangxi | SChina | Canine |
| GQ472477 | 2006 | Guangxi | SChina | Canine |
| GQ472478 | 2007 | Guangxi | SChina | Canine |
| AB573762 | 2006 | Philippines | Island1 | Human |
| AB573763 | 2006 | Philippines | Island1 | Human |
| HQ450385 | 2004 | Anhui | NChina | Canine |
| HQ118101 | 1989 | Anhui | NChina | Canine |
| HQ118102 | 2006 | Guizhou | SChina | Canine |
| HQ118103 | 2008 | Zhejiang | SChina | Canine |
| HQ118104 | 1989 | Anhui | NChina | Canine |
| HQ118105 | 2008 | Zhejiang | SChina | Canine |
| HQ118106 | 2008 | Zhejiang | SChina | Canine |
| HQ118107 | 2008 | Zhejiang | SChina | Canine |
| HQ118108 | 2008 | Zhejiang | SChina | Canine |
| HQ118109 | 2008 | Zhejiang | SChina | Canine |
| HQ118110 | 2008 | Zhejiang | SChina | Canine |
| HQ118111 | 2008 | Zhejiang | SChina | Canine |
| HQ118112 | 2008 | Zhejiang | SChina | Canine |
| HQ118114 | 2008 | Zhejiang | SChina | Ferret badger |
| HQ118115 | 2008 | Zhejiang | SChina | Ferret badger |
| HQ118116 | 2008 | Zhejiang | SChina | Ferret badger |
| HQ118117 | 2008 | Zhejiang | SChina | Ferret badger |
| HQ118118 | 2008 | Zhejiang | SChina | Ferret badger |
| GU994209 | 2009 | Zhejiang | SChina | Homo sapiens |
| HQ695732 | 2009 | Shaanxi | NChina | Canine |
| JN609295 | 2008 | Fujian | SChina | Canine |
| JQ423952 | 2011 | Beijing | NChina | Equine |
| JF819603* | 2008 | Yunnan | Yunnan | Canine |
| JF819604* | 2008 | Yunnan | Yunnan | Canine |
| JF819605* | 2008 | Yunnan | Yunnan | Canine |
| JF819606* | 2009 | Yunnan | Yunnan | Homo sapiens |
| JF819607* | 2009 | Yunnan | Yunnan | Homo sapiens |
| JF819608* | 2008 | Yunnan | Yunnan | Canine |
| JF819609* | 2008 | Yunnan | Yunnan | Canine |
| JF819610* | 2008 | Yunnan | Yunnan | Canine |
| JF819611* | 2009 | Yunnan | Yunnan | Canine |
| JF819612* | 2009 | Yunnan | Yunnan | Canine |
| JF819614* | 2009 | Yunnan | Yunnan | Homo sapiens |
| JF819615* | 2008 | Yunnan | Yunnan | Canine |
| JF819616* | 2009 | Yunnan | Yunnan | Canine |
| JF819617* | 2008 | Yunnan | Yunnan | Human |
| JF819618* | 2008 | Yunnan | Yunnan | Human |
| JF819619* | 2010 | Yunnan | Yunnan | Canine |
| JF819620* | 2010 | Yunnan | Yunnan | Canine |
| JF819621* | 2010 | Yunnan | Yunnan | Human |
| JF819622* | 2010 | Yunnan | Yunnan | Canine |
| JF819623* | 2010 | Yunnan | Yunnan | Canine |
| JF819624* | 2010 | Yunnan | Yunnan | Homo sapiens |
| JQ040591* | 2011 | Yunnan | Yunnan | Canine |
| JQ040592* | 2011 | Yunnan | Yunnan | Canine |
| JQ040593* | 2010 | Yunnan | Yunnan | Human |
| JQ040594* | 2011 | Yunnan | Yunnan | Canine |
| JQ040595* | 2010 | Yunnan | Yunnan | Human |
| JQ040596* | 2010 | Yunnan | Yunnan | Canine |
| JQ040597* | 2010 | Yunnan | Yunnan | Canine |
| JQ040598* | 2011 | Yunnan | Yunnan | Canine |
| JQ040599* | 2010 | Yunnan | Yunnan | Canine |
| JQ040600* | 2010 | Yunnan | Yunnan | Canine |
| JQ647510 | 2011 | Hubei | NChina | Donkey |
| JQ794536 | 2011 | Yunnan | Yunnan | Donkey |
| JQ794537 | 2011 | Yunnan | Yunnan | Canine |
| JQ794538 | 2011 | Yunnan | Yunnan | Canine |
| JQ794539 | 2011 | Yunnan | Yunnan | Canine |
| JQ794540 | 2011 | Yunnan | Yunnan | Mule |
| JQ794541 | 2011 | Yunnan | Yunnan | Canine |
| JQ798943 | 2011 | Anhui | NChina | Canine |
| JQ798944 | 2011 | Anhui | NChina | Canine |
| JQ798945 | 2011 | Anhui | NChina | Canine |
| JQ798946 | 2011 | Anhui | NChina | Canine |
| JQ798947 | 2011 | Anhui | NChina | Canine |
| JQ798948 | 2011 | Anhui | NChina | Canine |
| JQ798949 | 2011 | Anhui | NChina | Canine |
| JQ798950 | 2011 | Anhui | NChina | Canine |
| JQ798951 | 2011 | Anhui | NChina | Canine |
| JQ798952 | 2011 | Anhui | NChina | Canine |
| JQ798953 | 2011 | Anhui | NChina | Canine |
| JQ798954 | 2011 | Anhui | NChina | Canine |
| JQ798955 | 2011 | Anhui | NChina | Canine |
| JQ798956 | 2011 | Anhui | NChina | Canine |
| JQ798957 | 2011 | Anhui | NChina | Canine |
| JQ798958 | 2011 | Anhui | NChina | Canine |
| JQ798959 | 2011 | Anhui | NChina | Canine |
| JQ798960 | 2011 | Anhui | NChina | Canine |
| JQ798961 | 2011 | Anhui | NChina | Canine |
| JQ730682 | 2010 | Yunnan | Yunnan | Canine |
| JN974823 | 2008 | Guangdong | SChina | Canine |
| JN974824 | 2005 | Guangxi | SChina | Canine |
| JN974826 | 2006 | Guangxi | SChina | Canine |
| JN974827 | 2006 | Guangxi | SChina | Canine |
| JN974828 | 2006 | Guangxi | SChina | Canine |
| JN974829 | 2008 | Guangxi | SChina | Canine |
| JN974830 | 2005 | Guizhou | SChina | Canine |
| JN974831 | 2005 | Guizhou | SChina | Homo sapiens |
| JN974832 | 2009 | Guizhou | SChina | Homo sapiens |
| JN974833 | 2010 | Guizhou | SChina | Canine |
| JN974834 | 2010 | Guizhou | SChina | Canine |
| JN974835 | 2010 | Hebei | NChina | Homo sapiens |
| JN974836 | 2005 | Hunan | SChina | Canine |
| JN974837 | 2005 | Hunan | SChina | Canine |
| JN974838 | 2005 | Hunan | SChina | Canine |
| JN974839 | 2005 | Hunan | SChina | Canine |
| JN974840 | 2006 | Hunan | SChina | Canine |
| JN974841 | 2006 | Hunan | SChina | Homo sapiens |
| JN974842 | 2006 | Hunan | SChina | Canine |
| JN974843 | 2006 | Hunan | SChina | Homo sapiens |
| JN974844 | 2007 | Hunan | SChina | Canine |
| JN974845 | 2008 | Hunan | SChina | Canine |
| JN974846 | 2008 | Hunan | SChina | Canine |
| JN974847 | 2008 | Hunan | SChina | Homo sapiens |
| JN974848 | 2009 | Hunan | SChina | Canine |
| JN974849 | 2008 | Jiangsu | NChina | Homo sapiens |
| JN974850 | 2008 | Jiangsu | NChina | Canine |
| JN974851 | 2008 | Jiangsu | NChina | Canine |
| JN974852 | 2008 | Jiangsu | NChina | Canine |
| JN974853 | 2009 | Jiangxi | SChina | Canine |
| JN974854 | 2009 | Jiangxi | SChina | Canine |
| JN974855 | 2009 | Jiangxi | SChina | Canine |
| JN974856 | 2008 | Sichuan | NChina | Canine |
| JN974857 | 2008 | Sichuan | NChina | Canine |
| JN974858 | 2009 | Sichuan | NChina | Canine |
| JN974859 | 2009 | Sichuan | NChina | Canine |
| JN974860 | 2009 | Sichuan | NChina | Canine |
| JN974861 | 2010 | Sichuan | NChina | Canine |
| JN974862 | 2010 | Sichuan | NChina | Canine |
| JN974863 | 2010 | Sichuan | NChina | Canine |
| JN974864 | 2008 | Shandong | NChina | Canine |
| JN974865 | 2008 | Shandong | NChina | Canine |
| JN974866 | 2009 | Shandong | NChina | Canine |
| JN974867 | 2009 | Shandong | NChina | Canine |
| JN974868 | 2009 | Shandong | NChina | Canine |
| JN974869 | 2009 | Shandong | NChina | Canine |
| JN974870 | 2003 | Shanghai | SChina | Canine |
| JN974871 | 2003 | Shanghai | SChina | Canine |
| JN974872 | 2003 | Shanghai | SChina | Canine |
| JN974873 | 2004 | Shanghai | SChina | Canine |
| JN974874 | 2005 | Shanghai | SChina | Canine |
| JN974875 | 2005 | Shanghai | SChina | Canine |
| JN974876 | 2009 | Shanxi | NChina | Canine |
| JN974877 | 2008 | Zhejiang | SChina | Ferret badger |
| JN974878 | 2008 | Zhejiang | SChina | Canine |
| JQ950448 | 2012 | Jiangxi | SChina | Ferret badger |
| JQ950450 | 2012 | Jiangxi | SChina | Ferret badger |
| JQ950452 | 2012 | Jiangxi | SChina | Ferret badger |
| JX005929 | 2008 | Sichuan | NChina | Canine |
| JX005930 | 2010 | Sichuan | NChina | Canine |
| JX005931 | 2008 | Sichuan | NChina | Canine |
| JX005932 | 2008 | Sichuan | NChina | Canine |
| JX005933 | 2008 | Sichuan | NChina | Canine |
| JX005934 | 2008 | Sichuan | NChina | Canine |
| JX005935 | 2009 | Sichuan | NChina | Canine |
| JX005936 | 2010 | Sichuan | NChina | Canine |
| JX005937 | 2008 | Sichuan | NChina | Canine |
| JX005938 | 2008 | Sichuan | NChina | Canine |
| JX005939 | 2008 | Sichuan | NChina | Canine |
| JX005940 | 2009 | Sichuan | NChina | Canine |
| JX005941 | 2009 | Sichuan | NChina | Canine |
| JX005942 | 2009 | Sichuan | NChina | Canine |
| JX005943 | 2010 | Sichuan | NChina | Canine |
| JX005944 | 2009 | Sichuan | NChina | Canine |
| JX005945 | 2010 | Sichuan | NChina | Canine |
| JX005946 | 2010 | Sichuan | NChina | Canine |
| JX005947 | 2010 | Sichuan | NChina | Canine |
| JX088694 | 2012 | Guangdong | SChina | Pig |
| JN786877 | 2012 | Thailand | SEA | Canine |
| JX123685 | 2010 | Zhejiang | SChina | Canine |
| JX123686 | 2010 | Zhejiang | SChina | Canine |
| JX123687 | 2010 | Zhejiang | SChina | Homo sapiens |
| JX123688 | 2009 | Zhejiang | SChina | Homo sapiens |
| KC169986 | 2009 | Guangxi | SChina | Rabid cattle |
| JX276405* | 2010 | Yunnan | Yunnan | Canine |
| JX276406* | 2011 | Yunnan | Yunnan | Canine |
| JX276407* | 2012 | Yunnan | Yunnan | Canine |
| JX276408* | 2011 | Yunnan | Yunnan | Canine |
| JX276409* | 2012 | Yunnan | Yunnan | Canine |
| JX276410* | 2011 | Yunnan | Yunnan | Canine |
| JX276411* | 2012 | Yunnan | Yunnan | Human |
| JX276412* | 2012 | Yunnan | Yunnan | Canine |
| JX276413* | 2012 | Yunnan | Yunnan | Canine |
| JX276414* | 2012 | Yunnan | Yunnan | Canine |
| JX276415* | 2012 | Yunnan | Yunnan | Canine |
| JX276416* | 2012 | Yunnan | Yunnan | Canine |
| JX276417* | 2012 | Yunnan | Yunnan | Canine |
| JX276418* | 2012 | Yunnan | Yunnan | Canine |
| JX276419* | 2012 | Yunnan | Yunnan | Canine |
| JX276420* | 2012 | Yunnan | Yunnan | Canine |
| JX276421* | 2012 | Yunnan | Yunnan | Canine |
| JX276422* | 2012 | Yunnan | Yunnan | Homo sapiens |
| JX276423* | 2012 | Yunnan | Yunnan | Canine |
| JX276424* | 2012 | Yunnan | Yunnan | Canine |
| JX276425* | 2012 | Yunnan | Yunnan | Canine |
| KC762941 | 2009 | Jiangxi | SChina | Melogale moschata |
| KC660078 | 2012 | Beijing | NChina | Canine |
| KF501181 | 2013 | Taiwan | Island1 | Ferret badger |
| KF501182 | 2013 | Taiwan | Island1 | Ferret badger |
| KF501183 | 2013 | Taiwan | Island1 | Ferret badger |
| KF501184 | 2013 | Taiwan | Island1 | Ferret badger |
| KF501185 | 2013 | Taiwan | Island1 | Ferret badger |
| KF663511 | 2011 | Shaanxi | NChina | Canine |
| KF663512 | 2011 | Shaanxi | NChina | Canine |
| KF663513 | 2011 | Shaanxi | NChina | Canine |
| KF663514 | 2011 | Shaanxi | NChina | Canine |
| KF663515 | 2011 | Shaanxi | NChina | Canine |
| KF663516 | 2011 | Shaanxi | NChina | Canine |
| KF663517 | 2011 | Shaanxi | NChina | Canine |
| KF663518 | 2011 | Shaanxi | NChina | Canine |
| KF663519 | 2011 | Shaanxi | NChina | Canine |
| KF663520 | 2011 | Shaanxi | NChina | Canine |
| KF663521 | 2012 | Shaanxi | NChina | Canine |
| KF663522 | 2012 | Shaanxi | NChina | Canine |
| KF663523 | 2009 | Zhejiang | SChina | Canine |
| KF663524 | 2010 | Zhejiang | SChina | Canine |
| KF663525 | 2009 | Zhejiang | SChina | Canine |
| KF663526 | 2011 | Shaanxi | NChina | Canine |
| KF663527 | 2012 | Shaanxi | NChina | Canine |
| KF663528 | 2012 | Shaanxi | NChina | Homo sapiens |
| KF663529 | 2012 | Shaanxi | NChina | Canine |
| KF663530 | 2012 | Shaanxi | NChina | Canine |
| KF726852 | 2009 | Jiangxi | SChina | Ferret badger |
| KP881356 | 2013 | Taiwan | Island1 | Ferret badger |

* acquired in our surveillance

**Table S2** Time-annotated (near) full genome sequences used for estimating evolutionary rates

| Accession number | | | | | | | |
| --- | --- | --- | --- | --- | --- | --- | --- |
| KR534256 | LM645033 | KR906792 | KR906742 | FJ712193 | GU358653 | JQ685958 | JQ730682 |
| KR534254 | LM645032 | KR906791 | KR906741 | AB569299 | GU345748 | JQ685957 | JQ423952 |
| KR534253 | LM645031 | KR906790 | KR906740 | AB981664 | GU345747 | JQ685956 | JN609295 |
| KR534252 | LM645030 | KR906789 | KR906739 | AB981663 | GU345746 | JQ685955 | AB618036 |
| KR534251 | LM645029 | KR906788 | KR906738 | LC029889 | JX473841 | JQ685954 | AB618035 |
| KR534250 | LM645028 | KR906787 | KR906737 | KP723638 | JX473840 | JQ685953 | AB618034 |
| KR534249 | LM645027 | KR906786 | KR906736 | KC252633 | JX473839 | JQ685952 | AB618032 |
| KR534248 | LM645026 | KR906785 | KR906735 | KC193267 | JX473838 | JQ685950 | HQ317918 |
| KR534247 | LM645025 | KR906784 | KR906734 | KJ004416 | HQ450385 | JQ685948 | FJ866836 |
| KR534246 | LM645024 | KR906783 | KM594043 | KM016899 | JQ944709 | JQ685947 | FJ866835 |
| KR534245 | LM645023 | KR906782 | KM594042 | KF726853 | JQ944708 | JQ685946 | EU293116 |
| KR534244 | LM645022 | KR906781 | KM594041 | KF726852 | JQ944707 | JQ685945 | EU293115 |
| KR534238 | LM645021 | KR906780 | KM594040 | KC252634 | JQ944706 | JQ685944 | EU293113 |
| KR534237 | LM645020 | KR906779 | KM594039 | KC977995 | JQ944705 | JQ685943 | EU293111 |
| KR534236 | LM645019 | KR906778 | KM594038 | KF977826 | JQ944704 | JQ685942 | GU647092 |
| KR534235 | LM645018 | KR906777 | KM594037 | KF620489 | JQ685977 | JQ685941 | EU886636 |
| KR534234 | LM645017 | KR906776 | KM594036 | KF620488 | JQ685976 | JQ685940 | EU886635 |
| KR534233 | LM645016 | KR906775 | KM594035 | KF620487 | JQ685972 | JQ685938 | EU886634 |
| KR534232 | LM645015 | KR906774 | KM594034 | JQ970487 | JQ685951 | JQ685934 | EU886633 |
| KR534231 | EU311738 | KR906773 | KM594033 | JQ970486 | JQ685949 | JQ685933 | EU886632 |
| KR534230 | KX036367 | KR906772 | KM594032 | JQ970485 | JQ685939 | JQ685932 | EU886631 |
| KR534229 | KX036366 | KR906771 | KM594031 | JQ970484 | JQ685937 | JQ685931 | EF564174 |
| KR534228 | KX036365 | KR906770 | KM594030 | JQ970483 | JQ685936 | JQ685929 | EU643590 |
| KR534220 | KX036364 | KR906769 | KM594029 | JQ970482 | JQ685935 | JQ685925 | FJ913470 |
| KR534219 | KX036363 | KR906768 | KM594028 | JQ970481 | JQ685930 | JQ685923 |  |
| KR534218 | KX036362 | KR906767 | KM594027 | JQ970480 | JQ685928 | JQ685922 |  |
| KR534217 | KX036361 | KR906766 | KM594026 | HE802676 | JQ685927 | JQ685921 |  |
| LM645056 | KU198479 | KR906765 | KM594025 | HE802675 | JQ685926 | JQ685920 |  |
| LM645055 | KU198478 | KR906764 | KM594024 | KC171645 | JQ685924 | JQ685919 |  |
| LM645054 | KU198477 | KR906763 | KM594023 | KC171644 | JQ685914 | JQ685918 |  |
| LM645053 | KU198476 | KR906762 | KT728349 | KC171643 | JQ685912 | JQ685917 |  |
| LM645052 | KU198475 | KR906761 | KT728348 | KC595283 | JQ685908 | JQ685916 |  |
| LM645051 | KU198474 | KR906760 | KT336437 | KC595282 | JQ685906 | JQ685915 |  |
| LM645050 | KU198473 | KR906759 | KT336436 | KC595281 | JQ685896 | JQ685913 |  |
| LM645049 | KU198472 | KR906758 | KT336435 | KC595280 | JQ685892 | JQ685911 |  |
| LM645048 | KU198471 | KR906757 | KT336434 | KC762941 | JQ685975 | JQ685910 |  |
| LM645047 | KU198470 | KR906756 | KT336433 | KF155002 | JQ685974 | JQ685909 |  |
| LM645046 | KU198469 | KR906755 | KT336432 | KF155001 | JQ685973 | JQ685907 |  |
| LM645045 | KU198468 | KR906754 | KP997032 | KF155000 | JQ685971 | JQ685905 |  |
| LM645044 | KU198467 | KR906753 | KT006769 | KF154999 | JQ685970 | JQ685904 |  |
| LM645043 | KU198466 | KR906752 | GQ412744 | KF154998 | JQ685969 | JQ685903 |  |
| LM645042 | KU198465 | KR906751 | KM272192 | KF154996 | JQ685968 | JQ685902 |  |
| LM645041 | KU198464 | KR906750 | JN234411 | KC737850 | JQ685966 | JQ685901 |  |
| LM645040 | KU198463 | KR906749 | FJ959397 | AB635373 | JQ685965 | JQ685900 |  |
| LM645039 | KU198462 | KR906748 | KJ564280 | KC660078 | JQ685964 | JQ685899 |  |
| LM645038 | KU198461 | KR906747 | KR230090 | JQ647510 | JQ685963 | JQ685898 |  |
| LM645037 | KU198460 | KR906746 | KR230089 | KC196743 | JQ685962 | JQ685897 |  |
| LM645036 | KU523255 | KR906745 | FJ712196 | KC169986 | JQ685961 | JQ685895 |  |
| LM645035 | LN879481 | KR906744 | FJ712195 | JQ946087 | JQ685960 | JQ685894 |  |
| LM645034 | KU946961 | KR906743 | FJ712194 | AB699220 | JQ685959 | JQ685893 |  |

**Table S3** The sampling time distribution of the final dataset

| Year | 1974 | 1975 | 1981 | 1983 | 1984 | 1985 | 1986 | 1989 | 1990 | 1991 |
| --- | --- | --- | --- | --- | --- | --- | --- | --- | --- | --- |
| Num* | 1 | 1 | 1 | 1 | 2 | 1 | 1 | 1 | 5 | 3 |
| Year | 1992 | 1993 | 1994 | 1995 | 1996 | 1997 | 1998 | 1999 | 2001 | 2002 |
| Num | 3 | 2 | 2 | 1 | 1 | 2 | 1 | 4 | 5 | 5 |
| Year | 2004 | 2005 | 2006 | 2008 | 2009 | 2010 | 2011 | 2012 | 2013 | 2014 |
| Num | 5 | 5 | 5 | 5 | 5 | 5 | 5 | 4 | 5 | 3 |
| Year | 2015 |  |  |  |  |  |  |  |  |  |
| Num | 2 |  |  |  |  |  |  |  |  |  |

* Number of sequences used

**Table S4** Subsampled sequences used in phylogeographical analysis

| Accession number | Year | Location | Area | Host |
| --- | --- | --- | --- | --- |
| AY218996 | 1999 | Thailand | SEA | Canine |
| AY218997 | 1999 | Thailand | SEA | Canine |
| AY218998 | 1999 | Thailand | SEA | Canine |
| AY218999 | 1999 | Thailand | SEA | Human |
| AY219000 | 1999 | Thailand | SEA | Human |
| AY219001 | 1999 | Thailand | SEA | Human |
| AY219002 | 1999 | Thailand | SEA | Human |
| AB154208 | 2001 | Indonesia | Island2 | Cat |
| AB154209 | 2002 | Indonesia | Island2 | Cat |
| AB154210 | 2001 | Indonesia | Island2 | Cat |
| AB154211 | 2002 | Indonesia | Island2 | Cat |
| AB154212 | 2001 | Indonesia | Island2 | Cattle |
| AB154213 | 2001 | Indonesia | Island2 | Civet cat |
| AB154214 | 2001 | Indonesia | Island2 | Deer |
| AB154215 | 2001 | Indonesia | Island2 | Canine |
| AB154216 | 2002 | Indonesia | Island2 | Canine |
| AB154220 | 1997 | Indonesia | Island2 | Canine |
| AB154221 | 2000 | Indonesia | Island2 | Canine |
| AB154222 | 2001 | Indonesia | Island2 | Canine |
| AB154223 | 1997 | Indonesia | Island2 | Canine |
| AB154224 | 2002 | Indonesia | Island2 | Monkey |
| AB154225 | 2000 | Indonesia | Island2 | Canine |
| AB154226 | 2000 | Indonesia | Island2 | Canine |
| AB154227 | 2000 | Indonesia | Island2 | Canine |
| AB154228 | 2001 | Indonesia | Island2 | Canine |
| AB154229 | 2002 | Indonesia | Island2 | Canine |
| AB154230 | 2002 | Indonesia | Island2 | Canine |
| AB154231 | 2002 | Indonesia | Island2 | Canine |
| AB154232 | 2002 | Indonesia | Island2 | Canine |
| AB154233 | 1997 | Indonesia | Island2 | Canine |
| AB154234 | 2000 | Indonesia | Island2 | Canine |
| AB154235 | 2000 | Indonesia | Island2 | Canine |
| AB154237 | 2001 | Indonesia | Island2 | Canine |
| AB154238 | 2001 | Indonesia | Island2 | Canine |
| AB154239 | 2001 | Indonesia | Island2 | Canine |
| AB154240 | 2002 | Indonesia | Island2 | Canine |
| AB154241 | 1997 | Indonesia | Island2 | Canine |
| AB154242 | 2001 | Indonesia | Island2 | Tiger |
| AB154243 | 2002 | Indonesia | Island2 | Canine |
| AB178892 | 1985 | Thailand | SEA | Canine |
| AB178893 | 1985 | Thailand | SEA | Canine |
| AB178894 | 1985 | Thailand | SEA | Canine |
| AB178895 | 1977 | Thailand | SEA | Canine |
| AB178896 | 1963 | Thailand | SEA | Canine |
| DQ496219 | 2006 | Hunan | SChina | Pig |
| DQ515993 | 2006 | Hunan | SChina | Canine |
| DQ666287 | 2004 | Guangxi | SChina | Canine |
| DQ666289 | 2004 | Guizhou | SChina | Canine |
| DQ666291 | 2004 | Guizhou | SChina | Canine |
| DQ666294 | 2004 | Guizhou | SChina | Canine |
| DQ666296 | 2004 | Guizhou | SChina | Canine |
| DQ666297 | 2004 | Henan | NChina | Canine |
| DQ666298 | 2004 | Henan | NChina | Canine |
| DQ666299 | 2004 | Henan | NChina | Canine |
| DQ666300 | 2004 | Henan | NChina | Canine |
| DQ666302 | 2004 | Henan | NChina | Canine |
| DQ666304 | 2004 | Henan | NChina | Canine |
| DQ666306 | 2004 | Henan | NChina | Canine |
| DQ666308 | 2004 | Hunan | SChina | Canine |
| DQ666309 | 2004 | Hunan | SChina | Canine |
| DQ666312 | 2004 | Hunan | SChina | Canine |
| DQ666317 | 2004 | Hunan | SChina | Canine |
| DQ666318 | 2004 | Hunan | SChina | Canine |
| DQ666319 | 2004 | Hunan | SChina | Canine |
| DQ666321 | 2004 | Jiangsu | NChina | Canine |
| DQ866106 | 2003 | Guangxi | SChina | Canine |
| DQ866107 | 2003 | Guangxi | SChina | Canine |
| DQ866108 | 2003 | Guangxi | SChina | Canine |
| DQ866109 | 2003 | Guangxi | SChina | Canine |
| DQ866110 | 2004 | Guangxi | SChina | Canine |
| DQ866111 | 2000 | Guangxi | SChina | Canine |
| DQ866112 | 2004 | Guangxi | SChina | Canine |
| DQ866113 | 2003 | Guangxi | SChina | Canine |
| DQ866115 | 2003 | Guangxi | SChina | Canine |
| DQ866116 | 2003 | Guangxi | SChina | Canine |
| DQ866117 | 2004 | Guangxi | SChina | Canine |
| DQ866118 | 2005 | Guangxi | SChina | Canine |
| DQ866121 | 2005 | Guangxi | SChina | Canine |
| AB294349 | 2005 | Guangxi | SChina | Wild pig |
| AB294350 | 2004 | Guangxi | SChina | Canine |
| AB294351 | 2004 | Guangxi | SChina | Canine |
| AB294352 | 2003 | Guangxi | SChina | Canine |
| AB294355 | 2005 | Guangxi | SChina | Bovine |
| EF611081 | 2004 | Hubei | NChina | Buffalo |
| EU008919 | 2005 | Hunan | SChina | Canine |
| EU008922 | 2005 | Hunan | SChina | Canine |
| EU008923 | 2005 | Hunan | SChina | Canine |
| EU095330 | 2006 | Yunnan | Yunnan | Canine |
| EU086164 | 1999 | Myanmar | SEA | Canine |
| EU086165 | 1999 | Myanmar | SEA | Canine |
| EU086166 | 1999 | Myanmar | SEA | Canine |
| EU086167 | 1999 | Cambodia | SEA | Canine |
| EU086168 | 1998 | Cambodia | SEA | Canine |
| EU086169 | 1998 | Cambodia | SEA | Canine |
| EU086170 | 1997 | Cambodia | SEA | Canine |
| EU086171 | 1999 | Cambodia | SEA | Canine |
| EU086172 | 1998 | Cambodia | SEA | Canine |
| EU086173 | 1998 | Jiangsu | NChina | Canine |
| EU086175 | 1997 | Guangxi | SChina | Canine |
| EU086176 | 1992 | Shanghai | SChina | Human |
| EU086182 | 1994 | Guangxi | SChina | Canine |
| EU086183 | 1994 | Guangxi | SChina | Canine |
| EU086185 | 1992 | Chongqing | NChina | Human |
| EU086188 | 2004 | Jiangxi | SChina | Canine |
| EU086192 | 2003 | Indonesia | Island2 | Canine |
| EU086193 | 1999 | Laos | SEA | Canine |
| EU086194 | 2002 | Laos | SEA | Canine |
| EU086195 | 2002 | Laos | SEA | Canine |
| EU086200 | 1994 | Philippines | Island1 | Canine |
| EU086202 | 1994 | Philippines | Island1 | Canine |
| EU086203 | 2000 | Philippines | Island1 | Human |
| EU086204 | 2001 | Philippines | Island1 | Homo sapiens |
| EU086205 | 2004 | Philippines | Island1 | Homo sapiens |
| EU086206 | 1983 | Thailand | SEA | Human |
| EU086207 | 1983 | Thailand | SEA | Human |
| EU086208 | 1983 | Thailand | SEA | Human |
| EU086209 | 2001 | Vietnam | SEA | Canine |
| EU086210 | 2001 | Vietnam | SEA | Canine |
| EU267777 | 2007 | Hebei | NChina | Human |
| EU159368 | 2004 | Anhui | NChina | Canine |
| EU159380 | 2005 | Hubei | NChina | Canine |
| EU159382 | 2005 | Jiangsu | NChina | Canine |
| EU159384 | 2005 | Jiangsu | NChina | Canine |
| EU159385 | 1997 | Guangxi | SChina | Canine |
| EU159388 | 1992 | Chongqing | NChina | Canine |
| EU159390 | 1998 | Guangxi | SChina | Canine |
| EU159392 | 1992 | Shanghai | SChina | Human |
| EU159393 | 1992 | Shanghai | SChina | Canine |
| EU159394 | 2006 | Shanghai | SChina | Canine |
| EU159395 | 2006 | Guizhou | SChina | Canine |
| EU159397 | 2006 | Zhejiang | SChina | Canine |
| EU159399 | 1994 | Guangxi | SChina | Canine |
| EU159400 | 1989 | Anhui | NChina | Canine |
| EU159401 | 1989 | Anhui | NChina | Canine |
| EU275243 | 2006 | Yunnan | Yunnan | Canine |
| EU275244 | 2007 | Yunnan | Yunnan | Canine |
| EU275245 | 2007 | Yunnan | Yunnan | Canine |
| EU293111 | 1983 | Thailand | SEA | Human |
| EU293121 | 1983 | Thailand | SEA | Human |
| EU549783 | 2006 | Hebei | NChina | Canine |
| EU700031 | 2007 | Beijing | NChina | Homo sapiens |
| EU700032 | 2008 | Zhejiang | SChina | Human |
| EU828651 | 2007 | Hebei | NChina | Canine |
| EU828653 | 2007 | Guangdong | SChina | Canine |
| EU828655 | 2007 | Hebei | NChina | Canine |
| EU828657 | 2007 | Hebei | NChina | Canine |
| EU643590 | 2006 | Hunan | SChina | Canine |
| FJ561726 | 2008 | Fujian | SChina | Canine |
| FJ561728 | 2008 | Fujian | SChina | Canine |
| FJ598135 | 2008 | Zhejiang | SChina | Ferret badger |
| FJ712194 | 2008 | Zhejiang | SChina | Canine |
| FJ719751 | 2008 | Jiangxi | SChina | Ferret badger |
| FJ719755 | 2008 | Jiangxi | SChina | Ferret badger |
| FJ719760 | 2008 | Zhejiang | SChina | Canine |
| FJ866829 | 2007 | Fujian | SChina | Canine |
| FJ866830 | 2007 | Fujian | SChina | Canine |
| FJ866831 | 2007 | Fujian | SChina | Canine |
| FJ866836 | 2008 | Fujian | SChina | Canine |
| FJ594278 | 1997 | Guangxi | SChina | Canine |
| GQ303555 | 2009 | Thailand | SEA | Homo sapiens |
| GQ303556 | 2009 | Thailand | SEA | Canine |
| GU233765 | 2009 | Jiangxi | SChina | Ferret badger |
| GU345746 | 1992 | Chongqing | NChina | Canine |
| GU345747 | 1986 | Ningxia | NChina | Homo sapiens |
| GU345748 | 2006 | Shanghai | SChina | Canine |
| GU358653 | 1994 | Guangxi | SChina | Canine |
| GU647092 | 2008 | Jiangxi | SChina | Ferret badger |
| GU591790 | 2009 | Shaanxi | NChina | Canine |
| GU591792 | 2009 | Sichuan | NChina | Canine |
| GU992307 | 1983 | Thailand | SEA | Canine |
| HM756692 | 2008 | Hunan | SChina | Canine |
| HM486348 | 2005 | Jiangsu | NChina | Canine |
| HM486349 | 2006 | Jiangsu | NChina | Canine |
| HM486350 | 2006 | Jiangsu | NChina | Canine |
| HM486351 | 2006 | Jiangsu | NChina | Canine |
| HM486352 | 2006 | Jiangsu | NChina | Canine |
| HM486353 | 2005 | Jiangsu | NChina | Canine |
| HM486354 | 2006 | Jiangsu | NChina | Canine |
| HM486355 | 2004 | Shanghai | SChina | Canine |
| HM486356 | 2004 | Shanghai | SChina | Canine |
| HM486357 | 2003 | Shanghai | SChina | Canine |
| HM486358 | 2003 | Shanghai | SChina | Canine |
| HM486359 | 2003 | Shanghai | SChina | Canine |
| HM486360 | 2005 | Anhui | NChina | Canine |
| HM486361 | 2005 | Anhui | NChina | Canine |
| HM486362 | 2005 | Anhui | NChina | Canine |
| HM486363 | 2006 | Jiangsu | NChina | Canine |
| HM486364 | 2006 | Jiangsu | NChina | Canine |
| HM486365 | 2006 | Guangxi | SChina | Canine |
| HM486366 | 2005 | Guangxi | SChina | Canine |
| HM486367 | 2005 | Guizhou | SChina | Canine |
| HM486368 | 2005 | Guizhou | SChina | Canine |
| HM486369 | 2006 | Guizhou | SChina | Canine |
| HM486370 | 2005 | Guangxi | SChina | Canine |
| HM486371 | 2005 | Guizhou | SChina | Canine |
| HM486372 | 2006 | Guizhou | SChina | Canine |
| HM486374 | 2008 | Zhejiang | SChina | Canine |
| HM486376 | 2008 | Shandong | NChina | Canine |
| HM486377 | 2008 | Shandong | NChina | Canine |
| HM486378 | 2007 | Shandong | NChina | Canine |
| HM486379 | 2007 | Shandong | NChina | Canine |
| HM486380 | 2006 | Shandong | NChina | Canine |
| HM486381 | 2007 | Shandong | NChina | Canine |
| GQ472468 | 2007 | Guangxi | SChina | Canine |
| GQ472469 | 2007 | Guangxi | SChina | Canine |
| GQ472470 | 2007 | Guangxi | SChina | Canine |
| GQ472471 | 2007 | Guangxi | SChina | Canine |
| GQ472472 | 2007 | Guangxi | SChina | Canine |
| GQ472473 | 2007 | Guangxi | SChina | Canine |
| GQ472474 | 2007 | Guangxi | SChina | Canine |
| GQ472475 | 2005 | Guangxi | SChina | Canine |
| GQ472476 | 2006 | Guangxi | SChina | Canine |
| GQ472477 | 2006 | Guangxi | SChina | Canine |
| GQ472478 | 2007 | Guangxi | SChina | Canine |
| AB573762 | 2006 | Philippines | Island1 | Human |
| AB573763 | 2006 | Philippines | Island1 | Human |
| HQ450385 | 2004 | Anhui | NChina | Canine |
| HQ118101 | 1989 | Anhui | NChina | Canine |
| HQ118102 | 2006 | Guizhou | SChina | Canine |
| HQ118117 | 2008 | Zhejiang | SChina | Ferret badger |
| HQ118118 | 2008 | Zhejiang | SChina | Ferret badger |
| GU994209 | 2009 | Zhejiang | SChina | Homo sapiens |
| HQ695732 | 2009 | Shaanxi | NChina | Canine |
| JN609295 | 2008 | Fujian | SChina | Canine |
| JQ423952 | 2011 | Beijing | NChina | Equine |
| JF819603 | 2008 | Yunnan | Yunnan | Canine |
| JF819604 | 2008 | Yunnan | Yunnan | Canine |
| JF819605 | 2008 | Yunnan | Yunnan | Canine |
| JF819606 | 2009 | Yunnan | Yunnan | Homo sapiens |
| JF819607 | 2009 | Yunnan | Yunnan | Homo sapiens |
| JF819608 | 2008 | Yunnan | Yunnan | Canine |
| JF819609 | 2008 | Yunnan | Yunnan | Canine |
| JF819610 | 2008 | Yunnan | Yunnan | Canine |
| JF819611 | 2009 | Yunnan | Yunnan | Canine |
| JF819612 | 2009 | Yunnan | Yunnan | Canine |
| JF819614 | 2009 | Yunnan | Yunnan | Homo sapiens |
| JF819615 | 2008 | Yunnan | Yunnan | Canine |
| JF819616 | 2009 | Yunnan | Yunnan | Canine |
| JF819617 | 2008 | Yunnan | Yunnan | Human |
| JF819618 | 2008 | Yunnan | Yunnan | Human |
| JF819619 | 2010 | Yunnan | Yunnan | Canine |
| JF819620 | 2010 | Yunnan | Yunnan | Canine |
| JF819621 | 2010 | Yunnan | Yunnan | Human |
| JF819622 | 2010 | Yunnan | Yunnan | Canine |
| JF819623 | 2010 | Yunnan | Yunnan | Canine |
| JF819624 | 2010 | Yunnan | Yunnan | Homo sapiens |
| JQ040591 | 2011 | Yunnan | Yunnan | Canine |
| JQ040592 | 2011 | Yunnan | Yunnan | Canine |
| JQ040593 | 2010 | Yunnan | Yunnan | Human |
| JQ040594 | 2011 | Yunnan | Yunnan | Canine |
| JQ040595 | 2010 | Yunnan | Yunnan | Human |
| JQ040596 | 2010 | Yunnan | Yunnan | Canine |
| JQ040597 | 2010 | Yunnan | Yunnan | Canine |
| JQ040598 | 2011 | Yunnan | Yunnan | Canine |
| JQ040599 | 2010 | Yunnan | Yunnan | Canine |
| JQ040600 | 2010 | Yunnan | Yunnan | Canine |
| JQ647510 | 2011 | Hubei | NChina | Donkey |
| JQ794536 | 2011 | Yunnan | Yunnan | Donkey |
| JQ794537 | 2011 | Yunnan | Yunnan | Canine |
| JQ794538 | 2011 | Yunnan | Yunnan | Canine |
| JQ794539 | 2011 | Yunnan | Yunnan | Canine |
| JQ794540 | 2011 | Yunnan | Yunnan | Mule |
| JQ794541 | 2011 | Yunnan | Yunnan | Canine |
| JQ798944 | 2011 | Anhui | NChina | Canine |
| JQ798945 | 2011 | Anhui | NChina | Canine |
| JQ798947 | 2011 | Anhui | NChina | Canine |
| JQ798952 | 2011 | Anhui | NChina | Canine |
| JQ798953 | 2011 | Anhui | NChina | Canine |
| JQ798956 | 2011 | Anhui | NChina | Canine |
| JQ798957 | 2011 | Anhui | NChina | Canine |
| JQ798958 | 2011 | Anhui | NChina | Canine |
| JQ798959 | 2011 | Anhui | NChina | Canine |
| JQ798960 | 2011 | Anhui | NChina | Canine |
| JQ798961 | 2011 | Anhui | NChina | Canine |
| JQ730682 | 2010 | Yunnan | Yunnan | Canine |
| JN974823 | 2008 | Guangdong | SChina | Canine |
| JN974824 | 2005 | Guangxi | SChina | Canine |
| JN974826 | 2006 | Guangxi | SChina | Canine |
| JN974827 | 2006 | Guangxi | SChina | Canine |
| JN974828 | 2006 | Guangxi | SChina | Canine |
| JN974829 | 2008 | Guangxi | SChina | Canine |
| JN974830 | 2005 | Guizhou | SChina | Canine |
| JN974831 | 2005 | Guizhou | SChina | Homo sapiens |
| JN974832 | 2009 | Guizhou | SChina | Homo sapiens |
| JN974833 | 2010 | Guizhou | SChina | Canine |
| JN974834 | 2010 | Guizhou | SChina | Canine |
| JN974835 | 2010 | Hebei | NChina | Homo sapiens |
| JN974836 | 2005 | Hunan | SChina | Canine |
| JN974839 | 2005 | Hunan | SChina | Canine |
| JN974840 | 2006 | Hunan | SChina | Canine |
| JN974841 | 2006 | Hunan | SChina | Homo sapiens |
| JN974842 | 2006 | Hunan | SChina | Canine |
| JN974843 | 2006 | Hunan | SChina | Homo sapiens |
| JN974844 | 2007 | Hunan | SChina | Canine |
| JN974845 | 2008 | Hunan | SChina | Canine |
| JN974847 | 2008 | Hunan | SChina | Homo sapiens |
| JN974848 | 2009 | Hunan | SChina | Canine |
| JN974849 | 2008 | Jiangsu | NChina | Homo sapiens |
| JN974850 | 2008 | Jiangsu | NChina | Canine |
| JN974851 | 2008 | Jiangsu | NChina | Canine |
| JN974852 | 2008 | Jiangsu | NChina | Canine |
| JN974853 | 2009 | Jiangxi | SChina | Canine |
| JN974854 | 2009 | Jiangxi | SChina | Canine |
| JN974855 | 2009 | Jiangxi | SChina | Canine |
| JN974856 | 2008 | Sichuan | NChina | Canine |
| JN974857 | 2008 | Sichuan | NChina | Canine |
| JN974858 | 2009 | Sichuan | NChina | Canine |
| JN974859 | 2009 | Sichuan | NChina | Canine |
| JN974860 | 2009 | Sichuan | NChina | Canine |
| JN974861 | 2010 | Sichuan | NChina | Canine |
| JN974862 | 2010 | Sichuan | NChina | Canine |
| JN974863 | 2010 | Sichuan | NChina | Canine |
| JN974864 | 2008 | Shandong | NChina | Canine |
| JN974865 | 2008 | Shandong | NChina | Canine |
| JN974866 | 2009 | Shandong | NChina | Canine |
| JN974867 | 2009 | Shandong | NChina | Canine |
| JN974868 | 2009 | Shandong | NChina | Canine |
| JN974869 | 2009 | Shandong | NChina | Canine |
| JN974870 | 2003 | Shanghai | SChina | Canine |
| JN974871 | 2003 | Shanghai | SChina | Canine |
| JN974872 | 2003 | Shanghai | SChina | Canine |
| JN974873 | 2004 | Shanghai | SChina | Canine |
| JN974874 | 2005 | Shanghai | SChina | Canine |
| JN974875 | 2005 | Shanghai | SChina | Canine |
| JN974876 | 2009 | Shanxi | NChina | Canine |
| JN974877 | 2008 | Zhejiang | SChina | Ferret badger |
| JQ950448 | 2012 | Jiangxi | SChina | Ferret badger |
| JQ950450 | 2012 | Jiangxi | SChina | Ferret badger |
| JQ950452 | 2012 | Jiangxi | SChina | Ferret badger |
| JX005929 | 2008 | Sichuan | NChina | Canine |
| JX005930 | 2010 | Sichuan | NChina | Canine |
| JX005931 | 2008 | Sichuan | NChina | Canine |
| JX005932 | 2008 | Sichuan | NChina | Canine |
| JX005933 | 2008 | Sichuan | NChina | Canine |
| JX005934 | 2008 | Sichuan | NChina | Canine |
| JX005935 | 2009 | Sichuan | NChina | Canine |
| JX005936 | 2010 | Sichuan | NChina | Canine |
| JX005937 | 2008 | Sichuan | NChina | Canine |
| JX005938 | 2008 | Sichuan | NChina | Canine |
| JX005939 | 2008 | Sichuan | NChina | Canine |
| JX005940 | 2009 | Sichuan | NChina | Canine |
| JX005941 | 2009 | Sichuan | NChina | Canine |
| JX005942 | 2009 | Sichuan | NChina | Canine |
| JX005943 | 2010 | Sichuan | NChina | Canine |
| JX005944 | 2009 | Sichuan | NChina | Canine |
| JX005945 | 2010 | Sichuan | NChina | Canine |
| JX005946 | 2010 | Sichuan | NChina | Canine |
| JX005947 | 2010 | Sichuan | NChina | Canine |
| JX088694 | 2012 | Guangdong | SChina | Pig |
| JN786877 | 2012 | Thailand | SEA | Canine |
| JX123685 | 2010 | Zhejiang | SChina | Canine |
| JX123686 | 2010 | Zhejiang | SChina | Canine |
| JX123687 | 2010 | Zhejiang | SChina | Homo sapiens |
| JX123688 | 2009 | Zhejiang | SChina | Homo sapiens |
| KC169986 | 2009 | Guangxi | SChina | Rabid cattle |
| JX276405 | 2010 | Yunnan | Yunnan | Canine |
| JX276406 | 2011 | Yunnan | Yunnan | Canine |
| JX276407 | 2012 | Yunnan | Yunnan | Canine |
| JX276408 | 2011 | Yunnan | Yunnan | Canine |
| JX276409 | 2012 | Yunnan | Yunnan | Canine |
| JX276410 | 2011 | Yunnan | Yunnan | Canine |
| JX276411 | 2012 | Yunnan | Yunnan | Human |
| JX276412 | 2012 | Yunnan | Yunnan | Canine |
| JX276413 | 2012 | Yunnan | Yunnan | Canine |
| JX276414 | 2012 | Yunnan | Yunnan | Canine |
| JX276415 | 2012 | Yunnan | Yunnan | Canine |
| JX276416 | 2012 | Yunnan | Yunnan | Canine |
| JX276417 | 2012 | Yunnan | Yunnan | Canine |
| JX276418 | 2012 | Yunnan | Yunnan | Canine |
| JX276419 | 2012 | Yunnan | Yunnan | Canine |
| JX276420 | 2012 | Yunnan | Yunnan | Canine |
| JX276421 | 2012 | Yunnan | Yunnan | Canine |
| JX276422 | 2012 | Yunnan | Yunnan | Homo sapiens |
| JX276423 | 2012 | Yunnan | Yunnan | Canine |
| JX276424 | 2012 | Yunnan | Yunnan | Canine |
| JX276425 | 2012 | Yunnan | Yunnan | Canine |
| KC762941 | 2009 | Jiangxi | SChina | Melogale moschata |
| KC660078 | 2012 | Beijing | NChina | Canine |
| KF501181 | 2013 | Taiwan | Island1 | Ferret badger |
| KF501182 | 2013 | Taiwan | Island1 | Ferret badger |
| KF501183 | 2013 | Taiwan | Island1 | Ferret badger |
| KF501184 | 2013 | Taiwan | Island1 | Ferret badger |
| KF501185 | 2013 | Taiwan | Island1 | Ferret badger |
| KF663511 | 2011 | Shaanxi | NChina | Canine |
| KF663512 | 2011 | Shaanxi | NChina | Canine |
| KF663513 | 2011 | Shaanxi | NChina | Canine |
| KF663517 | 2011 | Shaanxi | NChina | Canine |
| KF663518 | 2011 | Shaanxi | NChina | Canine |
| KF663520 | 2011 | Shaanxi | NChina | Canine |
| KF663521 | 2012 | Shaanxi | NChina | Canine |
| KF663522 | 2012 | Shaanxi | NChina | Canine |
| KF663523 | 2009 | Zhejiang | SChina | Canine |
| KF663524 | 2010 | Zhejiang | SChina | Canine |
| KF663525 | 2009 | Zhejiang | SChina | Canine |
| KF663526 | 2011 | Shaanxi | NChina | Canine |
| KF663527 | 2012 | Shaanxi | NChina | Canine |
| KF663528 | 2012 | Shaanxi | NChina | Homo sapiens |
| KF663529 | 2012 | Shaanxi | NChina | Canine |
| KF663530 | 2012 | Shaanxi | NChina | Canine |
| KF726852 | 2009 | Jiangxi | SChina | Ferret badger |
| KP881356 | 2013 | Taiwan | Island1 | Ferret badger |
